# Spontaneous Cell Competition in Immortalized Mammalian Cell Lines

**DOI:** 10.1371/journal.pone.0132437

**Published:** 2015-07-22

**Authors:** Alfredo I. Penzo-Méndez, Yi-Ju Chen, Jinyang Li, Eric S. Witze, Ben Z. Stanger

**Affiliations:** 1 Departments of Medicine and Cell and Developmental Biology, Perelman School of Medicine at the University of Pennsylvania, Philadelphia, Pennsylvania, United States of America; 2 Department of Cancer Biology, Perelman School of Medicine at the University of Pennsylvania, Philadelphia, Pennsylvania, United States of America; 3 Abramson Family Cancer Research Institute, Perelman School of Medicine at the University of Pennsylvania, Philadelphia, Pennsylvania, United States of America; Spanish National Research Council (CSIC), SPAIN

## Abstract

Cell competition is a form of cell-cell interaction by which cells compare relative levels of fitness, resulting in the active elimination of less-fit cells, “losers,” by more-fit cells, “winners.” Here, we show that in three routinely-used mammalian cell lines – U2OS, 3T3, and MDCK cells – sub-clones arise stochastically that exhibit context-dependent competitive behavior. Specifically, cell death is elicited when winner and loser sub-clones are cultured together but not alone. Cell competition and elimination in these cell lines is caspase-dependent and requires cell-cell contact but does not require *de novo* RNA synthesis. Moreover, we show that the phenomenon involves differences in cellular metabolism. Hence, our study demonstrates that cell competition is a common feature of immortalized mammalian cells in vitro and implicates cellular metabolism as a mechanism by which cells sense relative levels of “fitness.”

## Introduction

Tissue growth is influenced by both systemic cues and local cell interactions. In Drosophila, cell competition is a well-described example of the latter type of interaction, in which the presence of growth-advantaged “winner” cells triggers apoptosis of otherwise viable, but growth-disadvantaged “loser” cells [1–3]. One remarkable feature of cell competition is that cellular responses are triggered by relative rather than absolute growth properties, indicating that cells are able to sense neighboring cell “fitness” and compare it to their own [1–3]. Several molecular pathways have been implicated in cell competition in Drosophila, including dMyc and its ribosomal targets [4–6], components of the Dlg/Lgl/Scrib cell polarity complex [7–9], the BMP pathway [10], the Hippo pathway, and the Wnt and JAK/STAT pathways [11, 12]. Differences in the activity of these signaling pathways across cells result in competition-mediated cell death, but the mechanisms involved remain poorly understood.

The first evidence for mammalian cell competition came from the study of the *Belly spot and tail* (*Bst*) mutation in the mouse. *Bst* impairs ribosomal biogenesis and cell growth, but heterozygous *Bst* blastocysts still develop into viable animals of normal size. Nonetheless, *Bst* blastocysts injected with wild-type embryonic stem (ES) cells grow into embryos derived mostly from the grafted cells, indicating that *Bst* cells are outcompeted during embryogenesis [13]. Two recent studies confirmed that differences in Myc expression drive cell competition in cultured ES cells as well as in the mouse epiblast [14, 15]. Cell competition has also been described in adult tissues, as embryonic hepatic progenitors grafted in adult livers expand at the expense of resident hepatocytes in an age-dependent manner [16, 17]. Moreover, knock-down of Scribbled results in loser behavior in immortalized epithelial Madin-Darby canine kidney (MDCK) cells [18, 19].

In this study, we sought to determine whether cell competition could be modeled *in vitro*. To this end, we generated sub-clones from commonly cultured cell lines and tested them in various combinations for competitive interactions. Here we report that such sub-clones display context-dependent apoptosis in the presence of the parental cell population. Elimination of loser cells requires direct contact with winners and does not involve transcriptional changes. Competitive behavior is abrogated by conditions that limit metabolic activity or uncouple ATP generation from oxidative phosphorylation, indicating that changes in energetic metabolism underlie cell “fitness.” Taken together, these data indicate that cell competition is active in cell lines grown under routine culture conditions, reflecting a conserved role in regulating cellular dynamics.

## Results

### Isolation of mammalian cell lines exhibiting context-dependent apoptosis

Cell competition has been shown to occur in the mouse epiblast in response to endogenous differences in cellular fitness, which are reflected by heterogenous Myc protein levels [14]. Based on this, we hypothesized that differences in cell fitness might also occur in immortalized cells grown under standard culture conditions, resulting in the spontaneous emergence of winner and loser cells *i*n vitro. To test this hypothesis, we transfected U2OS human osteosarcoma cells with vectors carrying histone 2B-green fluorescent protein (H2B-EGFP), H2B-Venus or H2B-mCherry fusion protein expression cassettes along with a neomycin resistance cassette followed by G418 (neomycin) selection to generate a large number of stable U2OS sub-lines, or clones. We then performed pairwise co-cultures of cells from each one of the clones and parental, “wild-type” (Wt) U2OS cells, using the presence of fluorescent markers in the “clone” cells to monitor the co-culture cell composition over time. We hoped in this way to identify clones that would be eliminated from the co-cultures (“losers”) or that would conversely eliminate the wild type cells and take over the culture (“winners”).

**Fig 1:**
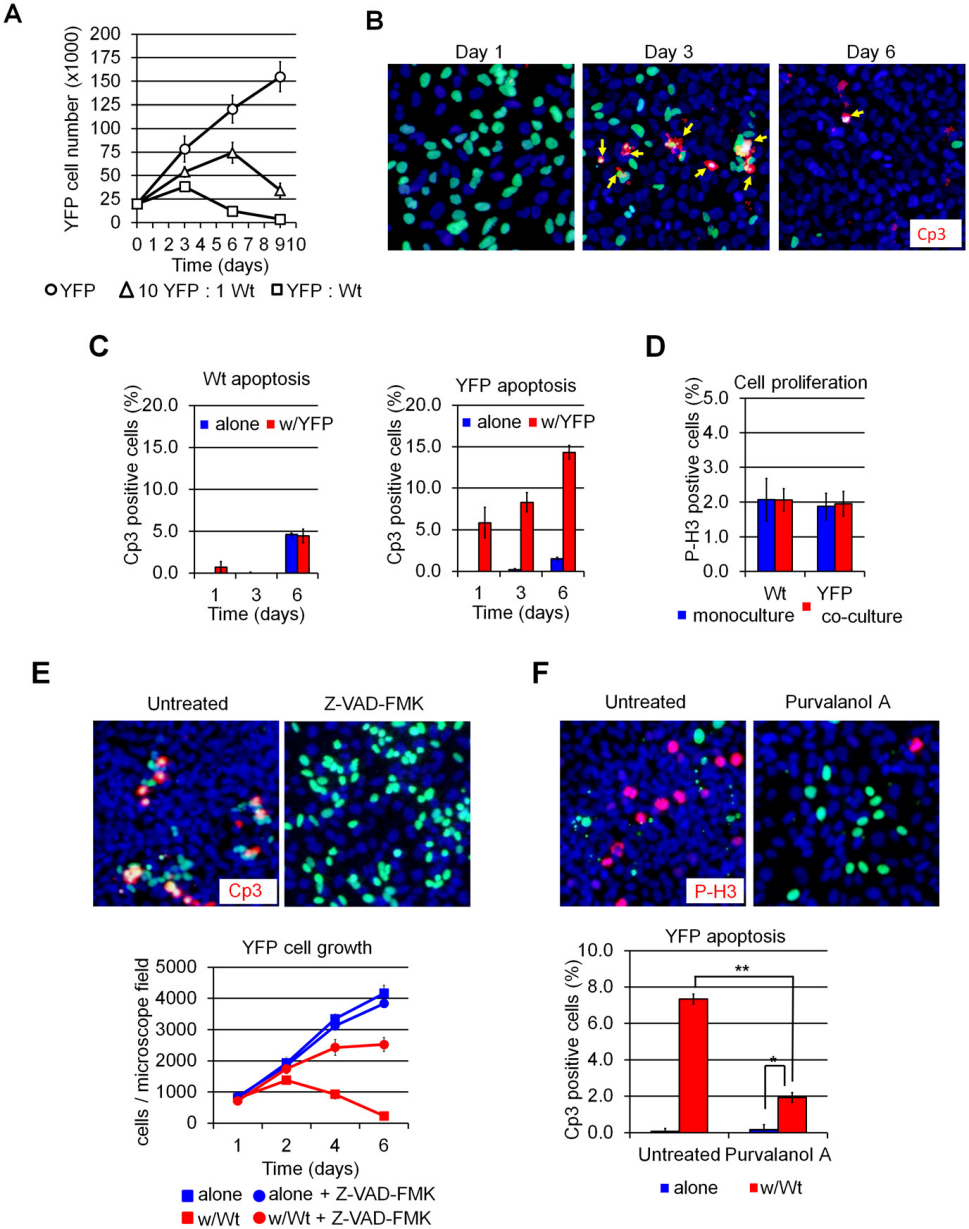
Cell death is triggered by a cell competition-like interaction in clonally-derived mammalian cell lines. (A) Cell counts showing YFP (“loser” cells) cells first expand, then decline, in the presence of Wt (“winner”) cells but grow unimpeded when cultured alone. Time is measured from cell seeding (t = 0). (B) Cleaved caspase-3 (Cp3) immuno-fluorescence (IF) (red) of U2OS cultures showing increased apoptosis in co-cultured YFP cells (green). Arrows indicate Cp3+ apoptotic YFP cells. Wt cells are counterstained with Hoescht 33342 (blue). (C) Quantification of apoptosis on immune-stained cultures; x-axis, time in days (d) Note that the baseline level of apoptosis increases with cell density by day 6 under all culture conditions. (D) Quantification of cell proliferation in 72-hour U2Os cultures by phospho-histone H3 (PH3) immunofluorescence. (E) Cp3 IF of 72-hour U2OS cultures treated with the caspase-3 inhibitor Z-VAD-FMK. YFP cell counts per microscope field are shown at the bottom. Inhibition of apoptosis by Z-VAD-FM K prevents YFP elimination from Wt:YFP co-cultures. (F) P-H3 IF of U2OS cells cultured for 72 hours in presence of the Cyclin D1 inhibitor purvalanol A as indicated. Quantification of apoptosis is shown below. Purvalanol A treatment inhibits proliferation (top) and rescues YFP elimination (bottom). Images were taken at 100X magnification. Error bars in this and all subsequent figures reflect mean ± SD. *: p<0.05, **: p<0.01 and ***: p<0.001 by Student’s *t*-test.

A small subset (9 out of 245) of the screened clones spontaneously exhibited a net *decrease* in their numbers upon co-culture with Wt cells (Fig 1A). Staining for cleaved caspase-3 (Cp3) confirmed that the cells were being eliminated by apoptosis (Fig 1B and 1C). No changes in cell proliferation were observed in co-cultured cells by phospho-histone H3 immunostaining (Fig 1D). Incubation of co-cultures with the pan-caspase inhibitor Z-VAD-FMK blocked cell elimination (Fig 1E). Furthermore, incubation with the CDK inhibitor purvalanol A also led to a dramatic decrease in killing, indicating that cells must be actively proliferating for cell death to occur (Fig 1F). When grown in mono-culture, growth rates varied widely among these “loser” clones, but all of them displayed substantially slower growth compared to that of the parental Wt cells (S1 Fig). Hence, U2OS cells maintained under routine conditions contain sub-clones that grow more slowly and are selectively killed when co-cultured with more rapidly-dividing cells, features reminiscent of cell competition in *Drosophila*. Notably, clones whose growth rate was similar to that of the parental Wt cells (e.g. clones G1 and 10A in S1 Fig) were not eliminated upon co-culture and did not show an increase in apoptosis. In addition, no clones growing faster than Wt cells or displaying “winner” behavior in co-culture were observed (data not shown).

To determine whether competition-like behavior is generalizable across different cell types from distinct species, we used the same strategy to screen randomly generated sub-clones from non-transformed murine fibroblasts (3T3 cells) and canine renal epithelial cells (MDCK cells). Consistent with our U2OS results, we found two MDCK clones (out of 169) and one 3T3 clone (out of 230) displaying increased apoptosis and cell elimination upon co-culture with parental cells (S2 and S3 Figs). Thus, competition-like behavior seems to be a general property of mammalian cell lines. We conducted all further experiments with U2OS cells.

### Elimination of U2OS losers requires direct contact with winners

**Fig 2:**
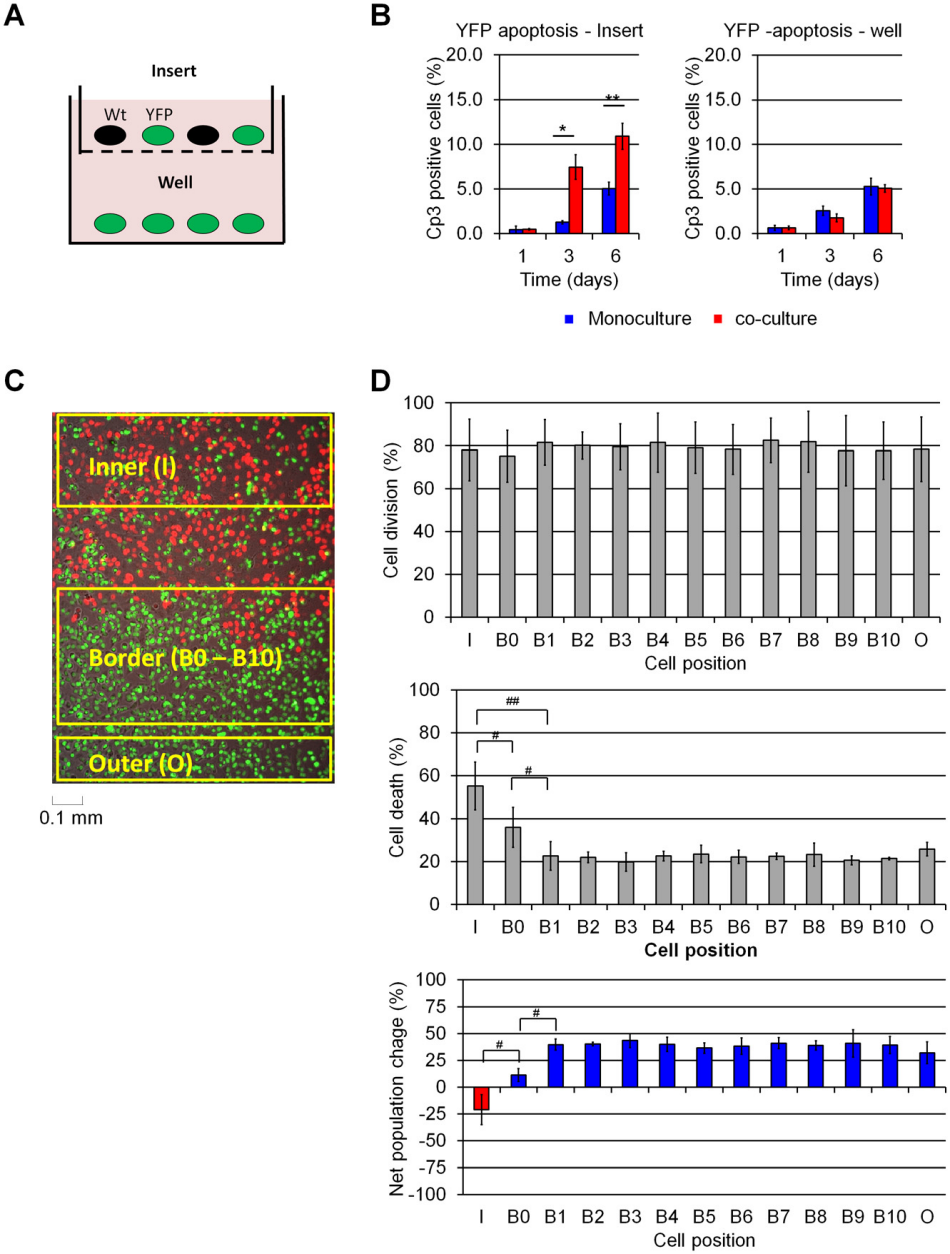
U2OS cell competition interactions are short-ranged. (A) Schematic representation of U2OS transwell cultures, Cells shared culture medium but were separated by a 0.8 μm Durapore membrane. (B) Cp3-IF analysis of apoptosis in transwell cultures. Wt cells induce apoptosis in YFP cells in the insert but not in YFP cells separated by the transwell. (C) Spot-seeding of YFP and H2B-mCherry expressing clone R1. Mixed YFP:R1 spots surrounded by pure YFP cell populations were divided in “inner” (I), “border” (B), and “outer” (O) zones as represented. (D) Time-lapse microscopy tracking of YFP cell fates during 72-hour spot cultures. The number of cell layers separating each YFP cell from its nearest R1 neighbor was recorded, and YFP “B” cells were grouped accordingly: for instance, a YFP cell is labeled “B3” if it comes within 3 cell layers of the nearest R1 cell, while a”B0” YFP cell comes to lie adjacent to an R1 cell at any time during the observation period. The data summarizes the fate of cells present at the beginning of each experiment and their immediate progeny, followed over 72 hours. The percentage of followed cells that underwent cell division is shown at the top; cell death is shown at the middle, and net population size change at the bottom. Increased apoptosis is observed only in inner YFP cells and “B0” border cells that come in direct contact with R1 cells. Data in panel D was derived from 3 independent experiments (Supplemental Movie S1-3), comprising over 5,000 cells counted. *: p<0.05, **: p<0.01 by Student’s *t*-test; #:p<0.05, ##:p<0.01 by paired *t*-test.

Cell competition in Drosophila has been reported to be mediated by soluble factors released when cells are in a competitive environment [4, 20]. To test whether cell competition in U2OS cells is also mediated by soluble factors, we performed a series of experiments utilizing trans-well filters and live cell imaging. For the trans-well experiments, we co-cultured YFP cells (which behave as losers) with Wt cells (which behave as winners) on the top of 0.8 μm Durapore trans-well inserts and placed YFP cells at the bottom of the well (Fig 2A). As expected, YFP cells in contact with Wt cells in the insert were eliminated (Fig 2B and 2C). By contrast, YFP cells in the well displayed no increase in apoptosis despite sharing the same medium with the Wt:YFP co-cultured cells in the insert (Fig 2B). Thus, cell competition in U2OS cells requires either direct contact or close proximity.

To distinguish between these possibilities, we examined cell interactions by time-lapse microscopy over a 72 h period (S4 Fig). In order to identify all cells in this experiment, we used R1 cells (red), which behave as winners when mixed with YFP cells (green). R1 cells were plated as a “spot” on tissue culture plastic and then overlaid with YFP cells; this created “Inner” and “Border” areas in which YFP and R1 cells were in frequent contact as well as an “Outer” region where YFP cells had no contact with R1 cells (Fig 2C and S4 Fig). We then tracked the fate of individual YFP to R1 cells, counting over 5,000 cells in 3 movies. As expected, YFP cells inside the R1 spot (“Inner” region) were progressively eliminated by apoptosis, while YFP cells that remained far from the R1 spot (>10 cell layers, “Outer” region) experienced significantly less apoptosis over the 72 h period analyzed (Fig 2D; S4 Fig and S1 File).

To more accurately determine the relationship between the fate of a cell and its location, we binned YFP cells based on their position relative to R1 cells, designating cells as “B0” to “B10” to reflect the smallest number of YFP cell layers observed between the designated cell and the nearest R1 cell. Relative cell position was estimated tracking the cell nuclei when cell contours could not be clearly seen (due to the high cell density reached by U2OS cultures). We observed that cell death was highly dependent upon designated cell position, with YFP cells in the immediate vicinity of R1 cells–“Inner” or “B0”–exhibiting significantly higher death rates than YFP cells that appeared to be even one cell removed from R1 cells. Indeed, B1 to B10 cells exhibited death rates similar to that observed in cells in the “Outer” region (Fig 2D, middle panel). Importantly, YFP cell proliferation was unaffected by position relative to R1 cells (Fig 2D, top panel). These rates of proliferation and death resulted in a net population decrease for cells in the “Inner” region and a net increase for all other cell populations (Fig 2D, bottom panel). These results indicate that YFP cell elimination is driven by direct contact with R1 cells. Furthermore, a stronger competitive response was observed in “inner” cells, in which losers were surrounded by winners, than in”B0” cells, in which losers might be in contact with only a single winner cell for only a brief period of time. This result suggests that loser cell elimination is dose-dependent, such that more extensive or longer contact with a winner cells increases the likelihood of the loser cell undergoing apoptosis.

### U2OS cell competition is context-dependent

Context-dependent responses—whereby cells behave as winners in the presence of less-fit clones, and as losers in the presence of more-fit clones—is a hallmark of cell competition in *Drosophila*. Because our screen for clones with varying fitness had only yielded cells that behaved as losers compared to Wt parental cells, we sought to determine whether this system exhibits context dependency.

**Fig 3:**
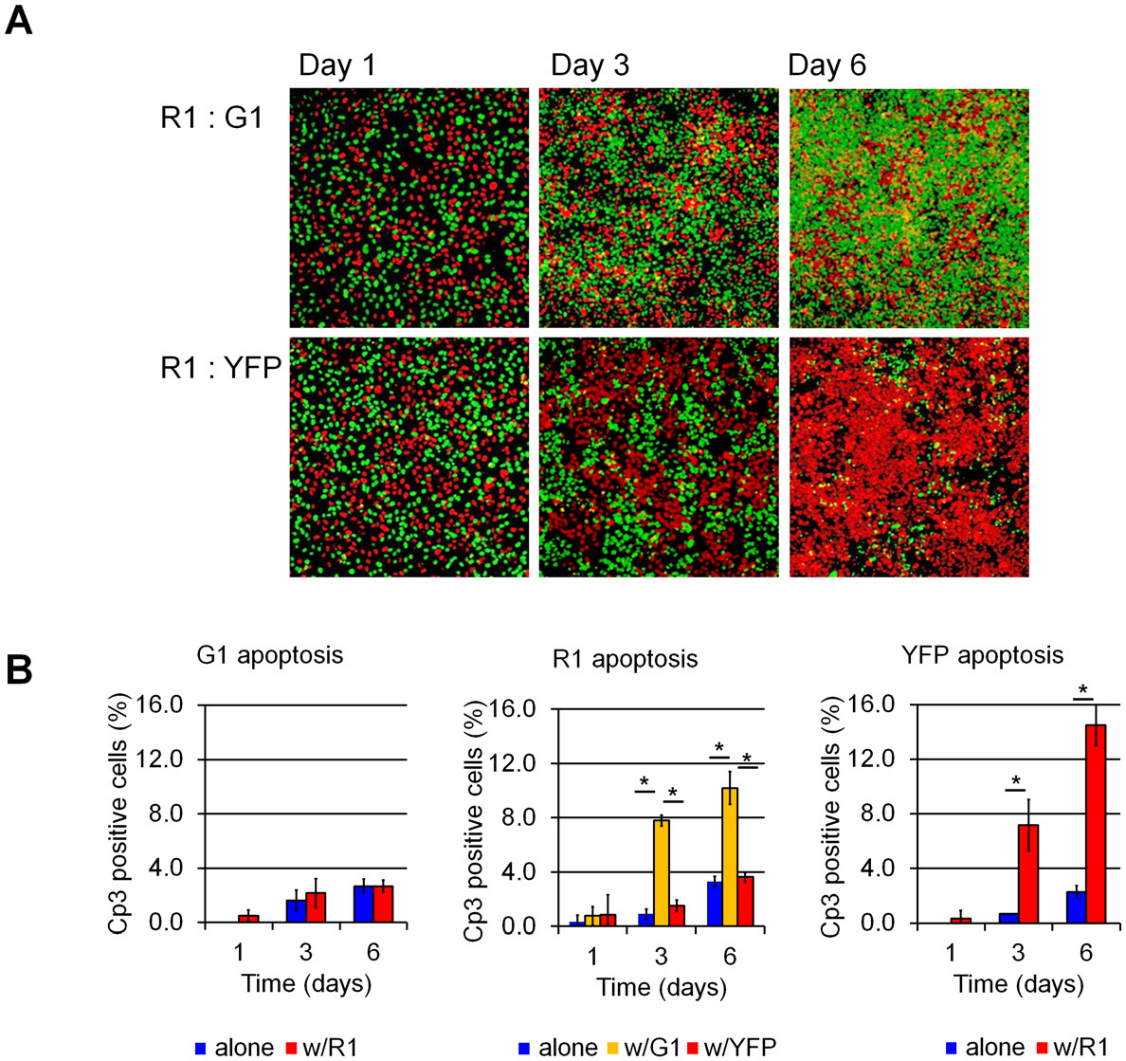
U2OS cell “fitness” is context-dependent. (A) Aspect of U2OS G1, R1, and YFP co-cultures in fluorescence microscopy. Cells were plated at a 1:1 ratio as indicated (R1:G1 or R1:YFP) and allowed to grow for 1, 3 or 6 days. (B) Cp3-IF apoptosis quantification. R1 cells behave as “winners” in R1:YFP cultures but as “losers” in the presence of G1 cells, indicating that R1 cells assume “winner” or “loser” status depending on the properties of their co-culture partners. *: p<0.001 by Student’s *t*-test.

We reasoned that R1 cells, which grow faster than some clones and slower than others (S1 Fig), would be good candidates for assessing whether U2OS competition is context-dependent. To this end, we co-cultured R1 cells with either G1 cells, which have a growth rate similar to Wt cells and do not compete with them (a “non-competing” clone), or with YFP cells, which grow more slowly. Co-culture of R1 and G1 cells led to a marked increase in R1 apoptosis, but no increase in G1 apoptosis (Fig 3A and 3B). Co-culture of R1 cells with three additional non-competing GFP clones yielded similar results (data not shown). By contrast, co-culture of R1 and YFP cells led to a marked increase in YFP apoptosis but no increase in R1 apoptosis (Fig 3A and 3B). Co-culture of the remaining GFP-expressing “loser” clones (S1 Fig) resulted in either non-competitive growth or the GFP-expressing cells still being eliminated (data not shown). Based on these results, we conclude that spontaneous cell competition in U2OS cells is context-dependent such that cells behave as winners or losers depending on the milieu in which they find themselves. Further pairwise assays demonstrated three “competition groups” in which higher proliferation rates and saturation densities were correlated with higher fitness (see color coding in S1 Fig). These data suggest that winner or loser status is not an intrinsic property of a cell, but is rather determined when cells confront their neighbors and relative levels of cell “fitness” are determined.

### U2OS cell competition does not require de novo RNA synthesis

In *Drosophila*, competitive fitness is affected by the activity of several genes, most notably Myc, the components of the Hippo pathway, and the members of the Scribd apico-basal polarity complex [4, 6–9, 21–24]. However, mosaic overexpression or knock-down of *MYC*, *YAP* and *SCRIB* did not alter cell competitive fitness in Wt U2OS cells (S5 and S6 Figs). Thus, competitive behavior in US02 cells is likely mediated by molecular mechanisms distinct from those that underlie such behavior in *Drosophila* S2 cells.

We reasoned that the determinants that confer relative level of fitness could reflect either a gain or a loss of information; if this were the case, then “loser status” might be expected to behave as either a dominant or a recessive trait, respectively. To address this idea, we carried out a series of cell fusion experiments in which heterokaryons were generated between various winner and loser U2OS clones. This fusion experiment resulted in diverse behaviors, with heterokaryons exhibiting winner status, loser status, or an intermediate phenotype depending on the identity of the parental clones (S7 Fig), suggesting that competitive “fitness” reflects the integration of multiple genetic or epigenetic factors.

**Fig 4:**
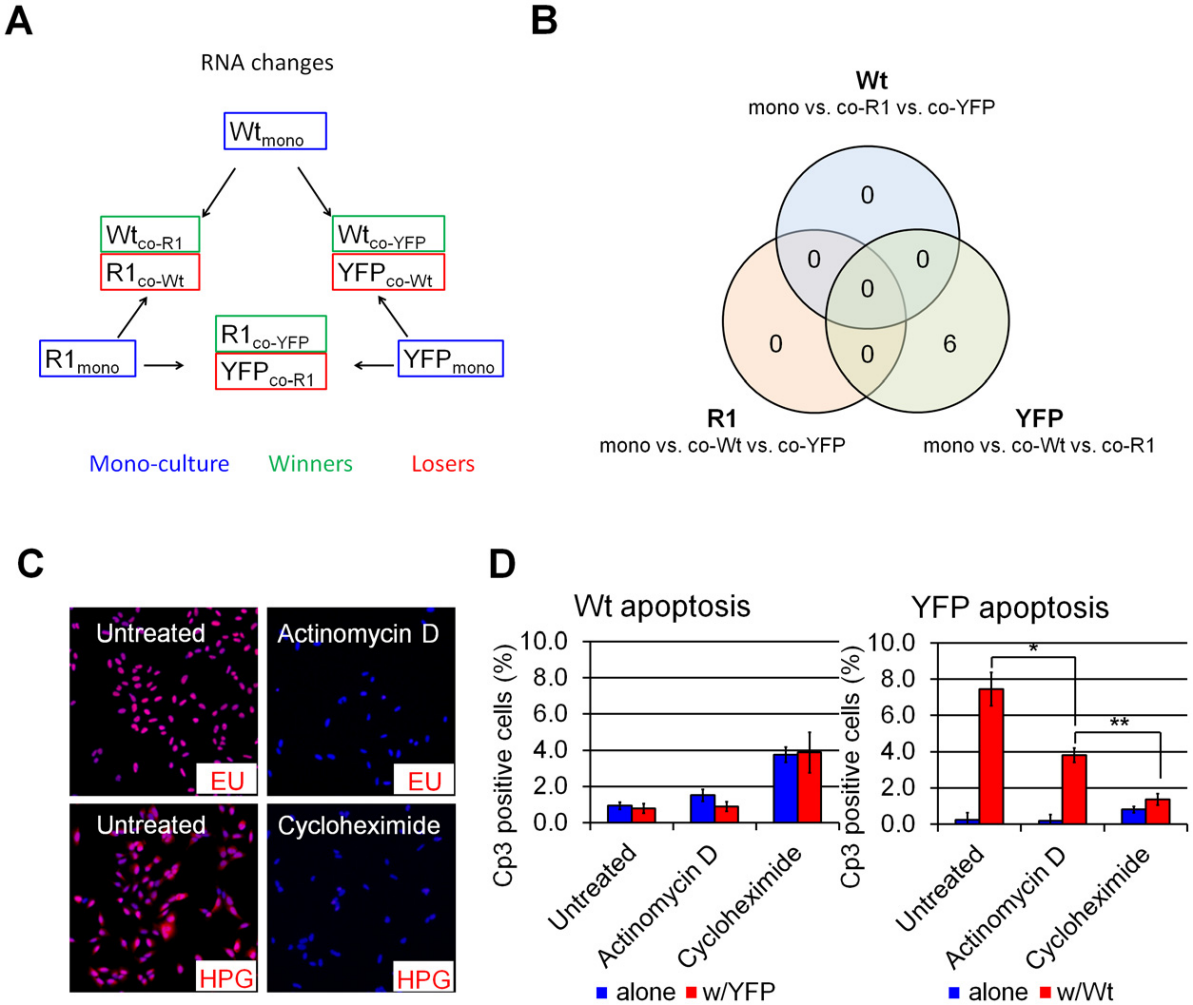
Cell competition in U2OS cells is mediated by post-transcriptional mechanism. (A) Microarray RNA analysis experimental design. Cells were grown in mono- or co-cultures for 48h as indicated and sorted by flow cytometry before RNA extraction. (B) Venn diagram distribution of RNAs displaying >2-fold expression change across indicated sample groups. Only six transcripts were found to be differentially expressed in mono-cultured and competing YFP cells, while no transcription changes were observed in response to competition in Wt and R1 cells. (C) Immuno-staining of metabolite-labeled nascent RNA and protein chains in Wt U2OS cells treated with actinomycin D or cycloheximide for 24 hours. Ethynil-uridine (EU) and homopropargyl-glycin (HGP) were added to label nascent RNA and peptide chains 2 h before staining. Complete inhibition of transcription and translation was obtained with actinomycin D and cycloheximide, respectively. (D) Cp3-IF of Wt:YFP 72-hour cultures grown in presence or absence of actinomycin D- and cycloheximide. Cycloheximide completely abolished Wt-induced YFP apoptosis. Actinomycin D treatment resulted in a partial rescue to a degree consistent with its inhibition of cell division (S10 Fig). *”p<0.05, **: p<0.01 by Student’s *t*-test.

We next compared the transcriptional profiles of Wt, YFP, and R1 cells in an attempt to identify genes driving cell competition. We expected that transcripts acting as fitness determinants would be differentially expressed in mono-cultured cells, most likely in a way that reflects their relative fitness (i.e., Wt>R1>YFP or vice versa). On the other hand, transcripts implicated in sensing or responding to fitness determinants might not be expected to differ in abundance in mono-cultures, but instead be differentially expressed only in actively-competing loser and winner cells (Fig 4A).

U2OS RNA expression levels were characterized by whole-transcriptome RNA microarray hybridization and compared between mono-cultured or competing Wt, R1 or YFP cells (Fig 4A). Unexpectedly, no transcriptional changes were found to be induced by competitive co-culture in Wt and R1 cells. Six transcripts were found to be expressed at higher levels in YFP cells when co-cultured with Wt cells. Of these, three were also up-regulated in YFP cells co-cultured with R1 cells (Fig 4B and S1 Table). However, the nature of these transcripts (encoding for the olfactory receptor 51B4, the interleukin 13 receptor subunit A2, and the gametocyte specific factor 1), and the fact that their expression was unchanged in loser R1 cells strongly suggest that changes observed in YFP cells are not related to competition.

Overall, these results suggested that competitive interactions do not spur unique transcriptional changes, raising the possibility that competition does not require the synthesis of new transcripts. To address this possibility, we blocked RNA and protein synthesis in Wt:YFP co-cultures using actinomycin D and cycloheximide, respectively. Despite a complete blockade in transcription following actinomycin D treatment (Fig 4C), YFP cells continued to be killed in the presence of Wt cells (Fig 4D). This result demonstrates that U2OS cell competition does not require de novo transcription. By contrast, cycloheximide treatment abolished the difference in apoptosis levels of control and co-cultured YFP cells (Fig 4D), indicating that cell competition requires protein translation. However, cycloheximide treatment also led to a proliferative arrest (S8 Fig), raising the possibility that cycloheximide blocks cell competition as a secondary consequence of its inhibition of cell cycle progression.

### Differences in metabolism underlie U2OS cell competition

We next turned our attention to potential fitness determinants. Comparison of transcriptional profiles across pooled mono- and co-cultured samples identified 656 genes that were differentially expressed in Wt, R1 and YFP cells. Of these, only 82 were expressed across Wt, R1, and YFP in a way correlating with cell fitness (S9 Fig) and gene ontology term representation analysis indicated no significant functional class enrichment except for the presence of 7 metalloprotease-encoding genes in the group (S2 Table). Importantly, further analysis of gene expression across the full panel of winners and losers (S1 Fig) failed to support the correlation in expression levels seen with Wt, R1, and YFP (data not shown). Thus, even among those transcripts which were differentially expressed between winners and losers, this transcriptional analysis did not lead to the emergence of strong candidates that might constitute determinants, sensors, or mediators of cell fitness and competition.

**Fig 5:**
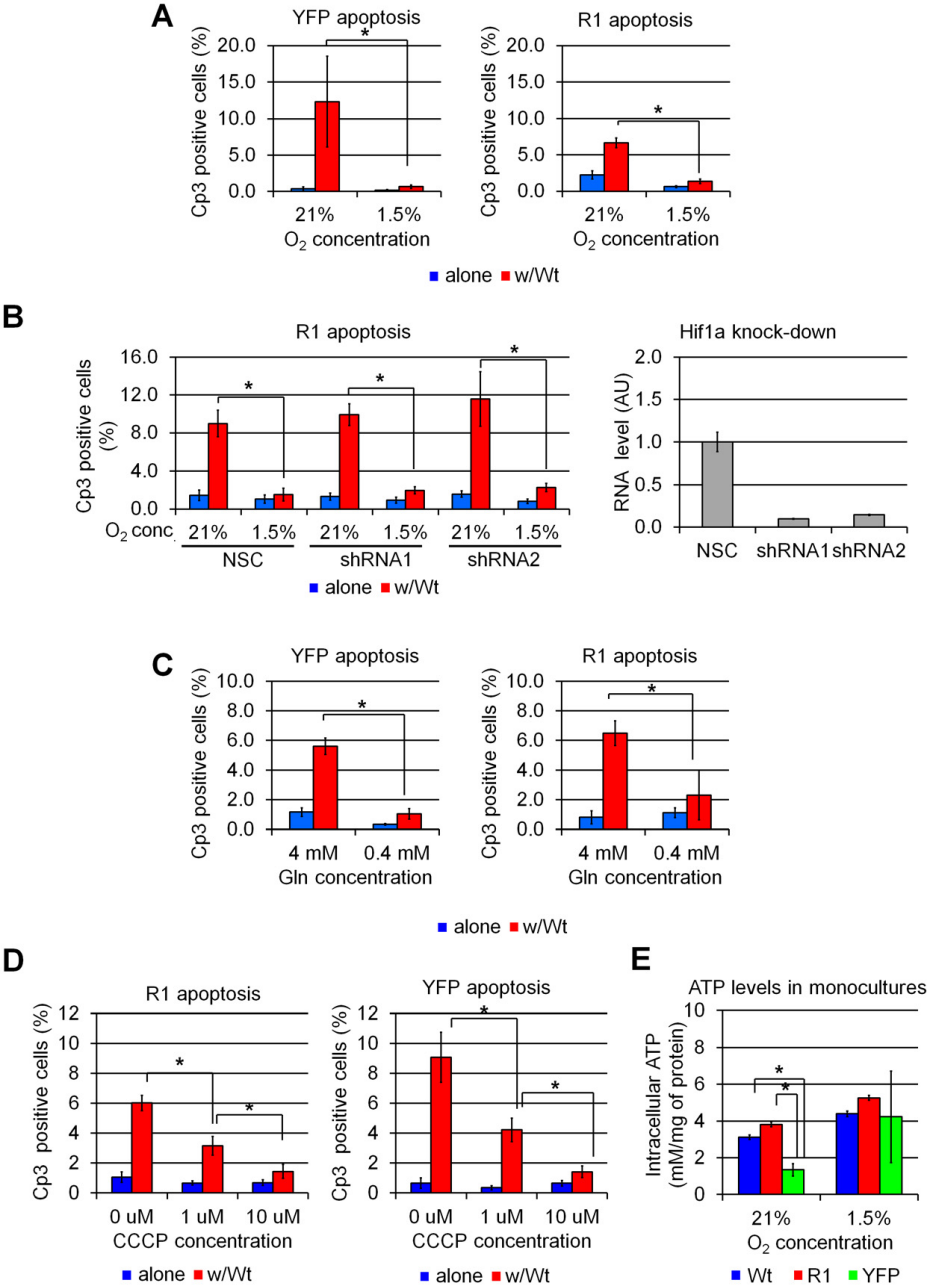
Differences in energy metabolism drive cell competition in mammalian cells. (A) Quantification of apoptosis (Cp3 immunofluorescence) in U2OS cultures grown for 72 hours under normoxic (21% O_2_) or hypoxic (1.5% O_2_) conditions. Hypoxia inhibits cell competition-induced elimination of YFP and R1 cells in Wt:YFP and Wt:R1 co-cultures. (B) Quantification of Cp3 immunofluorescence in U2OS R1 cells expressing a nonsense (NSC, non-silencing control) shRNA or shRNAs directed against the Hif1a transcription factor. Hif1a knockdown in loser cells does not affect cell competition. qPCR analysis of Hif1a in shRNA-expressing YFP cells is shown on the right. (C) Quantification of Cp3 immunofluorescence in U2OS cultures grown for 72 hours in medium containing standard (4 mM) and reduced (0.4 mM) Glutamin (Gln) concentrations. Withholding Gln arrests cell competition in U2OS cells. (D) Quantification of Cp3 immunofluorescence in U2OS cultures treated with a mitochondrial uncoupling agent (carbonyl cyanide m-chlorophenyl hydrazine, CCCP). Uncoupling respiration from oxidative phosphorylation blocks competition-induced elimination of R1 and YFP cells, indicating that competition is driven by differences in the activity of ATP-generating pathways. (E) Luciferase analysis of intracellular ATP levels in monocultured U2OS cell. YFP, but not R1 cells, display reduced ATP levels in when compared to Wt cells. Hypoxia increases ATP levels YFP cells, suggesting that reduced ATP levels may reflect or underlie YFP cell fitness.*: p<0.001, by one-way ANOVA.

Monopolization of trophic factors by metabolically advantaged “winners” was among the first mechanisms proposed to underlie cell competition. To test whether U2OS cells compete for trophic factors, we performed competition assays under culture conditions in which we varied the degree to which such factors might be limiting. Increasing serum concentration to 20% had no effect on loser cell elimination; nor did reducing or removing serum altogether, indicating that U2OS cells do not compete for serum-borne factors (S10 Fig). By contrast, hypoxia was found to completely block cell competition when either YFP or R1 cells were co-cultured with Wt cells (Fig 5A). As many of the effects hypoxia are promoted via activation of the Hif1a transcription factor [25], we sought to determine whether Hif1a was required for the oxygen dependency of U2OS cell competition. To this end, we performed the cell competition experiment in the presence of two different shRNAs directed against Hif1a or a non-silencing shRNA control (NSC). Hif1a knockdown did not protect YFP and R1 cells from cell competition under normoxia, nor did it reverse the protective effect of hypoxia (Fig 5B). These results strongly suggest that the ability of hypoxia to block cell competition is Hif1a-independent. These results were unexpected, as we had predicted that limiting amounts of O_2_ would exacerbate, rather than inhibit, cell competition.

Because hypoxia has several cellular effects—including changes in cell metabolism through effects on oxidative phosphorylation (OXPHOS) and reactive oxygen species (ROS) production—we hypothesized that differences in metabolic rate might underlie competitive behavior. Consistent with this notion, reducing the metabolic activity of cells by reducing the concentration of glutamine (Gln) in the medium eliminated cell competition in U2OS cells (Fig 5C). We then tested if competitive behavior might involve the production of ROS by carrying out competition assays in the presence of two ROS scavenging agents: N-acetyl cysteine (NAD) and 6-hydroxy-2,5,7,8-tetramethylchroman-2-carboxylic acid (Trolox). Neither NAD nor Trolox prevented loser cell elimination in U2OS co-cultures, indicating that ROS are dispensable for this process (S11 Fig).We next considered the possibility that ATP production (via OXPHOS) could be involved in cell competition in U2OS cells. To test this idea, U2OS competition assays were carried out in presence of carbonyl cyanide m-chloro phenyl hydrazine (CCCP), an agent that permeabilizes the inner mitochondrial membrane to H^+^ ions. CCCP thus allows OXPHOS to proceed but uncouples it from ATP synthesis. CCCP treatment resulted in a dose-dependent reduction in cell death when either YFP or R1 cells were co-cultured with Wt cells (Fig 5D). We reasoned that if ATP production underlies cell fitness, ATP levels in monocultured U2OS clones should reflect their fitness. Accordingly, intracellular ATP levels in monocultured YFP (loser) cells are lower than those observed in Wt (winner) and R1 (intermediate) cells. Furthermore, ATP concentration increased to near-Wt levels in YFP cells grown under hypoxia, correlating with the previous observation that it arrests Wt:YFP competition. Interestingly, R1 cells—the intermediate fitness cells—displayed comparative ATP levelswith WT (winner) under normxia and hypoxia. It suggests that ATP levels are not the sole metabolic product to determine cell fitness (Fig 5E). Taken together, these results strongly suggest that differences in energetic metabolism drive cell competition in U2OS cells and that production of ATP by OXPHOS is one of the parameters to determinate cell fitness.

## Discussion

Over the past 10 years, the phenomenon of cell competition has emerged as a mechanism by which cell growth and viability are controlled in diverse biological processes, such as tissue size regulation, aging, and cancer progression. Although most studies of cell competition have focused on Drosophila, a growing body of evidence indicates that the process is conserved in mammals [13–19, 26]. Nevertheless, the functional role and underlying molecular mechanisms of cell competition remain obscure. The results presented here show that mammalian cell lines spawn sub-clones that are viable and grow well on their own, but which undergo apoptosis in the presence of more advantaged cells. Relative cell fitness—the likelihood that a cell will behave as a winner or a loser—is at least partially associated with growth rate and saturation density and is context-dependent. This behavior is thus highly similar to the phenomenon of cell competition as it has been described in the Drosophila imaginal disc [4, 6, 27] and more recently in the embryonic mouse epiblast [14, 15].

Our results provide several clues about the determinants of cell completion in mammalian cells lines. At the cellular level, competition between U2OS cells was found to be mediated by short-range interactions, as only those loser cells located in the immediate vicinity of winners experienced increased apoptosis. Furthermore, our results suggest that cell competition involves dose-dependent interactions, since loser cells surrounded by winners had a greater likelihood of being eliminated than losers having winners on only one flank. These results differ somewhat from previous studies, in which cell competition was found to be mediated by diffusible factors when driven by Myc in *Drosophila* S2 cells [20] and by BMP deficiency in cultured mouse ES cells [15]. However, medium conditioned by competing mouse ES cells with different Myc expression levels does not elicit a response in naïve, loser cells [14]. Similarly, cell competiton resulting from mosaic inactivation of the cell polarity gene Scribbled in Madin-Darby canine kidney cells also requires direct winner-loser cell contact. *In vivo*, apoptosis resulting from cell competition occurs mostly within a few cell diameters of the winner-loser interface in the *Drosophila* imaginal disc [4, 6, 10, 28], the mouse epiblast,[14] and the mouse liver [16]. These results are all consistent with the notion that distinct cues mediate competitive interactions in different cell types, organisms, and stages of differentiation.

Two mechanisms have been proposed to describe how extracellular determinants could trigger competitive cell responses in the Drosophila imaginal disc [29]. In the first, cells compete for a limited supply of one or more factors required for survival. Similarly, competition could result from different cell sensitivities to toxins that accumulate in the microenvironment as cells grow [10]. Several observations argue against a mechanism of this type in the U2OS cells described in this study. Serum deprivation or enrichment did not affect cell competition while hypoxia and glutamine deprivation arrested it, indicating that none of these factors is limiting for growth. Alternatively, competition could involve a struggle for a limited amount of space on the substrate, in which winner cells displace less-adherent loser cells, thereby resulting in anoikis. Arguing against this possibility, however, time-lapse microscopy showed that losers undergo apoptosis before detaching from the substrate, a result inconsistent with an anoikis-based process. Morevover, YFP loser cells can attach and spread even when overlaid over confluent R1 winners, which is inconsistent with R1 cells physically displacing YFP cells. Finally, competition in U2OS cells is active at cell densities 5 to 10 fold below those reached by winners or losers at the end of their growth in monoculture.

A second mechanism involves a process by which cells sense and compare relative levels of “fitness.” One example of such a fitness-sensing mechanism is the role in imaginal disc cell competition played by the *flower* (Fwe) gene. Upon competition, alternative splicing results in expression of the Fwe^LoseA/B^ isoform in “loser” cells, which is necessary and sufficient for competition-driven cell elimination [30]. Furthermore, expression of mFwe1 –a mammalian homologue of *flower*–in the fly confers “loser” status in imaginal disc cells, suggesting a conserved role in competition [31]. In our study, no changes in the expression of the human mFwe1 homolog (FLOWER isoform 1) were observed in response to cell competition or across mono-cultured high and low-fitness clones, suggesting that cell competition in U2OS cells is *flower*-independent

Our data therefore point to a novel mechanism of cell competition in U2OS cells—one that is mediated by in a way that is independent of de novo RNA synthesis. Instead, our results suggest that differences in cell metabolism underlie the differences between winner and loser status. We found that hypoxia, which promotes a switch from OXPHOS to fermentation, arrests cell competition. Similarly, depriving cells of the non-essential amino acid Gln—a key fuel for the citric acid cycle—blocks cell competition. These results are thus in line with the recent study of de la Cova et al. [32], who showed that differences in energetic metabolism—mediated by Myc and p53 and resulting in a shift in the balance of glycolysis and OXPHOS—are key determinants of cell competition in Drosophila. In addition, we found that uncoupling OXPHOS from ATP production also arrests cell competition, indicating that differences in steady-state ATP levels and/or ATP production rates can drive cell competition. Monocultured YFP display decreased intracellular ATP levels compared to Wt cells, but ATP levels in YFP increase when cells are grown under hypoxia, suggesting that ATP levels may be a determinant of fitness in YFP cells. However, we did not observe similar ATP level changes in R1 cells. A possible explanation is that ATP levels are result of cell fitness rather than a determinant; R1 levels could be normal in monoculture but decrease upon competition. Alternatively, other products of energy metabolism could be involved in cell fitness determination. Further studies will be needed to characterize the role of energy metabolism in cell fitness, for instance by using living imaging techniques to monitor cell metabolism during cell competition.

In summary, we found that competitive behavior is a general property of common mammalian cell lines, supporting the notion that this phenomenon plays a role in maintaining the growth properties of mammalian cell populations by weeding out “unfit” cells. One interesting feature of our study is that all isolated clones behaved as losers when combined with the parental population; no spontaneous “supercompetitors” were isolated. This suggests that the process of immortalization and culture selects for populations of cells which exhibit a high fitness level at steady-state. Hence, our observations may have implications for cancer, in which tumor evolution may be driven in part by the eradication of less-fit cells through such interactions.

## Materials and Methods

### Cell culture

U2OS cells (American Tissue and Cell Collection, HTB-96) were a kind gift of Dr. John Hogenesh (University of Pennsylvania) NIH-3T3 (CRL-1658), MDCK (CCL-34) and 293T cells (CRL-3216) were directly obtained from ATCC. All cell lines were cultured in 10% Dulbecco’s modified Eagle medium supplemented to 10% decomplemented fetal bovine serum at 37°C, 5% CO_2_, 21% O_2_ and 100% humidity unless otherwise indicated. For aminoacid depletion experiments, dialyzed FBS was used and L-glutamine was supplemented as indicated. Cell lines were maintained and passaged according to ATCC recommended procedures. Pharmacological agents was as follows: Purvalanol-A (Sigma P4484), Z-VAD-FMK (EMD Millipore 627610), actinomycin D (Sigma A9415), cycloheximide (Sigma C7698), NAC (Sigma A9165), Trolox (Santa Cruz Biotech sc-200810) and CCCP (Sigma C2759) were added to cell cultures 12 h after cell seeding at the concentrations indicated and maintained until analysis, changing the medium every 24 hours. Ethynyl-uridine (Life Technologies E10345) and homopropargyl-glycine (Life Technologies C10186) were added at 5 and 50 μM respectively, two hours prior to incorporation analysis using the Click-iT alkyne detection kit (Life Technologies C10330).

### Transfection and selection of stable transfectant clones

Transfection was carried out using Fugene6 (Promega E2691). Selection of stable transfectant clones was obtained with G418 sulfate at 1 mg/ml (U2OS) or 0.5 mg/ml (3T3, MDCK),or hygromycin B at 150 μg/ml as appropriate. Stable transfectant clones were individually recovered from the selection plates using a fine pipette, amplified and cultured under standard conditions.

### Cell fusion

Heterokaryons were obtained by treating confluent cells with 50% PEG 1500 in 75 mM HEPES pH 8.0 for 1 minute followed by G418 + neomycin selection for 3 days. In contrast to stable transfection, individual clones were not separated after cell fusion, Heterokaryon sub-lines thus consisted of mixed populations resulting from many independent fusion events. Heterokaryon sub-lines were passage 3 times before using them in cell competition assays.

### Lentiviral transduction of single shRNAs

Cells were seeded at ~70% confluence and grown for 18 h before adding concentrated viral particles (described below), and further grown for 72 h. Selection of infected shRNA-exressing cells was then carried out using puromycin (5 μg/ml) for 48 h.

### Growth curves

For U2OS clone growth characterization, cells were seeded at a density of 6,600 cells/cm^2^. For U2OS, MDCK and 3T3competition assays, cells were seeded at 66 x 10^5^ cells/cm^2^, which results in 95% confluence after attachment and spreading. Cell seeding was considered t = 0 for all experiments and medium was changed every 48 hours. At indicated timepoints, cells were trypsinized and counted using an Accuri C6 flow cytometer. For the Z-VAD-FMK apoptosis inhibition experiments, cells were counted manually in 3 independent 10X microscope field.

### Cell death assays

Cells were seeded at 66,000 cells/cm2 (t = 0h) as mono-cultures or 1:1 co-cultures, which results in ~95% confluence after cell spreading in U2OS, 3T3 and MDCK cells. Apoptosis was analyzed after 1, 3 or 6 days in culture or as otherwise indicated by immunostaining with a cleaved (Asp175) Caspase-3 rabbit polyclonal antibody (Cell Signaling technologies 9661). Cleaved Caspase3-positive cells were counted on three independent 10X microscope fields for each sample and averaged. Growth curves were obtained by counting total cell numbers using an Accuri C6 cytometer. All assays were carried out in triplicate, and statistical significance of cell count comparisons was determined by Student’s t test.

### Time-lapse microscopy

U2OS R1 cells were spot-seeded at 10^6^ cells/ml on glass-bottom culture dishes (Mat-Tek Corporation), allowed to attach, and overlaid with YFP cells (10^6^ cells/ml). Cells were maintained at 37°C, 0.5% CO_2_ and 100% humidity while filmed at 15 min./frame using a Leica DM6000 B videomicroscope.

### Whole-transcriptome expression analysis

U2OS cells were grown as mono- or co-cultures for 48 h and separated on a FACSAria cell sorter (BD biosciences). RNA was isolated using the RNeasy kit (Qiagen) and hybridized to Affymatrix Human Gene 1.0 ST arrays at the Perelman School of Medicine Molecular Profiling Core (University of Pennsylvania). Each experimental condition was carried out in triplicate. Greater than 2-fold RNA expression changes were identified by 2-way (sample, replicate) ANOVA. Statistical analysis and hierarchical clustering was carried out using Partek Genomic Suite software (Partek Inc.). Gene ontology enrichment analysis was performed using the Gene Set Analysis Toolkit V2 (Gene Ontology Consortium). Microarray data are available in the ArrayExpress database (www.ebi.ac.uk/arrayexpress) under accession number E-MTAB-3204.

### Plasmids

H2B-YFP (Venus), H2B-EGFP, and H2B-mCherry sequences were obtained from Addgene (20971, 11680 and 20972, respectively) and cloned into pcDNA3.1-Neo^r^ or pcDNA3.1-Hygro^r^ backbones. These pCMV-H2B-XFP plasmids were used in U2OS YFP, G1-3 and R1 cells. For U2OS, 3T3 and MDCK clone screens, the promoter of pCMV-H2B-EGFP was replaced with the Ubiquitin-C promoter (pUB-GFP, Addgene 11155). The LSL-Myc and LSL-Yap plasmids were created in pcDNA 3.1 using a LoxP-(3x pSV40 polyA)-LoxP cassette, the Myc and Yap sequences were coded in-frame with the picornavirus 2A self-cleaving peptide and the H2B-EGFP sequences in order to allow bi-cistronic MYC/YAP and GFP expression. pMC-Cre has been described [33], and was obtained from Dr. Eric Brown (University of Pennsylvania).

### shRNA lentiviral vectors and viral particle preparation

pGIPZ shRNAs against Hif1a (shRNA1, RHS4430-98513964; shRNA2, RHS4430-101518881), YAP (RHS4430-98525388), Myc (RHS4430-98853488), Scribbled (RHS4430-98901643) and a non-silencing control (NSC, RHS4348) clones are part of the pGIPZ human whole transcriptome shRNA library (GE Dharmacon) and were kindly provided by Dr. Patrick Paddison (Fred Hutchinson Cancer Center). Lentiviral particles were obtained in 293T cells using the pSPAX2 (Addgene 12260) and pMD2.G (Addgene 12259) packing vector, following the standard procedures. For each lentiviral preparation, 60 x 10^6^ cells were transfected and cultured for 72 hours. Lentiviral particles in the medium were then precipitated using the Lenti-X concentrator reagent (Clontech 631231) and reconstituted in 1 ml of complete medium.

### qPCR

Myc, YAP, and Scribbled expression was analyzed using the Superscript RT kit (Life Technologies 18080–051) and the SSoadvanced SYBRGreen qPCR mix (BioRad 172–5260) with the following primers pairs: MYC (GTAGTGGAAAACCAGCAGCCT, AGAAATACGGCTGCACCGAG); YAP (TGACCCTCGTTTTGCCATGA, GTTGCTGCTGGTTGGAGTTG) Scribbled (AGGAGATCTACCGCTACAG, GATCTCAGGGATATCGTTCC); HIF1A (GTGAAGACATCGCGGGGA, GTGGCAACTGATGAGCAAGC); GAPDH (GGTGAAGGTCGGAGTCAACGG, GAGGTCAATGAAGGGGTCATTG).

### ATP assay

Cells were grown in 12-well plates as monocultures for 72 hours under normoxic (21% O_2_) or hypoxic (1.5% O_2_) conditions. Intracellular ATP was extracted by boiling water method as described previously by Yang et al. [34] with modification. Briefly, cells in 12-well plates were washed with cold PBS twice followed by adding 1 ml of boiling water to the wells directly. After repeated pipetting, cell extracts were cleared by centrifugation at 12000g for 5 min at 4°C. ATP levels in the supernatants were measured using the ENLITEN ATP Assay System Bioluminescence Detection Kit (Promega) following the manufacturer’s instructions. Total protein concentration was determined using the DC Protein Assay (BioRad) following the manufacturer’s recommended protocol.

## Supporting Information

S1 Fig Correlation between growth and fitness in U2OS cell clones. Growth curves of U2OS clones identified in the U2OS screen. Clones are grouped according to the outcome of co-culture with Wt, R1, and YFP cells (see Fig 3). High-fitness clones are shown in blue. They do not compete with Wt and behave as winners in the presence of R1. Clones shown in black behave as losers in the presence of Wt and do not compete with R1. Low-fitness clones, in red, behave as loser in presence of either Wt or R1.
(TIF)

S2 Fig Spontaneous cell competition in MDCK cells. (A) Growth curves of wild-type MDCK cells and H2B-GFP transfectant, single-cell derived 10D2 clone in mono- or co-culture. (B) Apoptosis quantification by Cp3-IF. 10D2 cells undergo increased apoptosis resulting in 10D2 cell number decrease in 10D2:Wt co-cultures.
(TIF)

S3 Fig Spontaneous cell competition in 3T3 cells. (A) Growth curves of wild-type 3T3 fibroblasts and H2B-GFP stable transfectant, single cell derived 13A5 clone cells in mono- or co-culture. (B) Apoptosis quantification by Cp3-IF. 13A5 cells are viable in mono-culture but display increased apoptosis in the presence of Wt cells and are progressively eliminated from the co-cultures.
(TIF)

S4 Fig Localized cell competition in U2OS-YFP:R1 spot cultures. (A) Schematic representation of spotted U2OS-R1 cells overlaid with U2OS-YFP cells. (B) Time-course micrographies of spot cultures. Detail of areas labeled as “1” and “2” is presented in (C) and (D), respectively. Progressive elimination of U2OS-YFP cells inside the R1 spot can be observed, but the spot boundary remains in place, indicating that YFP cells outside are still increasing in number.
(TIF)

S5 Fig Myc and YAP overexpression does not turn U2OS cells into supercompetitors. (A) Schematic representation of the inducible LSL-Myc-GFP expression cassette. Cre-mediated recombination results in excision of the SV40 polyadenylation signal (pA), placing the Myc-2A-GFP coding sequence under control of the CMV-actin hybrid promoter (Caggs). The Myc and GFP sequences are separated by the picornavirus 2A self-cleaving sequence, resulting in bi-cistronic MYC and GFP expression. The LSL-YAP-GFP expression cassette was similarly constructed by replacing the Myc coding sequence with that of the constitutive YAP^S117A^ mutant. (B) Aspect of LSL-Myc-GFP and LSL-YAP-GFP stable transfectant U2OS cells transiently transfected with a Cre expression vector (pMC-Cre). Cp3 IF quantification of apoptosis is shown in (C). Apoptosis rates in untransfected cells is similar or lower than that observed in Cre-transfected, Myc/YAP-GFP expressing cells, indicating that Myc and YAP expression does not confer supercompetitor status to U2OS cells.
(TIF)

S6 Fig Inactivation of Myc, YAP, or Scribbled does not result in competition in U2OS cells. (A) Growth curves of lentivirus-transduced U2OS cells expressing shRNAs directed against Myc, YAP, or Scribbled; cultured alone or alongside Wt cells. shRNA-expresing cells are recognized by means of a GFP expression cassette contained in the lentiviral shRNA vector (not shown). None of these shRNAs induced cell competition. (B) qPCR analysis of gene expression showing reduced Myc, YAP, and Scribbled RNA levels in shRNA-expressing cells. *: p<0.001 (Student’s *t*-test).
(TIF)

S7 Fig U2OS cell fitness is determined by intrinsic, dose-dependent determinants. (A) U2OS “winner-loser” cell fusion experimental design. Single-cell derived, H2B-mCherry, hygro^r^ stable transfectant clones (RH) were fused to YFP (neo^r^) cells to generate RY cell lines. RY cell fitness levels were then tested by co-culturing RY cells with either Wt or YFP cells. (B) Fluorescence micrographs and flow cytometry profiles of U2OS RY1 cells 4 days after fusion. The result is typical of observed cell fusion outcomes. Separate images of RY1 cell mCherry and YFP fluorescence are shown on the far right. Flow cytometry profiles showing YFP and mCherry co-expression in >95% of RY1 cells are displayed at the bottom. (C) Cp3 IF apoptosis analysis in 72 hour Wt, RH, RY and YFP cultures. Results shown are representative of 3 independent fusion experiments for each RH:YFP pairing. All three RH cell lines behave as “winners” in the presence of YFP cells. RH1:YFP fusion results in a fully “winner” line indicating that YFP “loser” status is rescued and therefore results from loss of function. However, RH4:YFP results in partial rescue and RH7:YFP fusion results in no rescue at all, suggesting that multiple factors determine U2OS cell “fitness”.
(TIF)

S8 Fig Expression levels across U2OS-Wt, R1, and YFP correlate with “fitness” level in a small subset of genes. (A) Venn diagram distribution of transcripts displaying a 2-fold or greater expression level difference in Wt vs. R1 vs.YFP cells by whole transcriptome microarray analysis (see Fig 4 for detail of experimental design). (B) Hierarchical clustering of expression profiles of the 422 transcripts displaying a greater than 2-fold expression level change in the Wt vs YFP comparison. Three clusters matching the profile Wt>R1>YFP and 2 clusters matching YFP>R1>Wt were identified (a total of 82 transcripts), suggesting that these transcripts could play a role as “fitness” determinants.
(TIF)

S9 Fig Transcription inhibition reduces proliferation in U2OS cells. (A) PH3 IF (red) of 48 h U2OS cell cultures grown in presences or absence of actinomycind D or cycloheximide as indicated. Actinomycin D treatment partially inhibits proliferation, suggesting that partial rescue of YFP cell elimination in treated Wt:YFP cultures is due to decreased overall culture growth (See Fig 5). Cycloheximide treatment results in complete proliferation blockade within 48 hours, along with cell competition arrest (Fig 5).
(TIF)

S10 Fig U2OS cell competition does not involve serum-borne factors. Cp3 IF analysis of apoptosis in 72 hour U2OS cultures grown in medium supplemented with fetal bovine serum at the concentrations indicated. Serum concentration does not affect apoptosis rates in R1 and YFP cells cultured alone or in alongside with Wt cells.
(TIF)

S11 Fig U2OS cell competition does not involve production of radical oxygen species (ROS). Cp3 IF analysis of apoptosis in 72 h U2OS cultures treated with raical ROS scavengers N-acetyl cysteine (NAC) (A) and 6-hydroxy-2,5,7,8-tetramethylchroman-2-carboxylic acid (Trolox) (B), as indicated. ROS scavengers do not reduce loser cell apoptosis rates in Wt:R1 or Wt:YFP co-cultures. A slight increase is observed in competing R1 cells at the highest Trolox concentration. *:p<0.05 (Student’s *t*-test).
(TIF)

S1 File U2OS cell competition is mediated by short-range cell interactions. Time-lapse microscospy video of R1:YFP spot co-cultures over 72 hours (1 frame = 15 min). Note that YFP (green nuclei) are progressively eliminated from the R1 (red nuclei) spot; however, the YFP cells outside the spot are not eliminated. Also note that in most cases death of YFP cells in the R1 spot is not preceded by cell detachment.
(MP4)

S1 Table RNA expression changes mono-cultured vs. competing U2OS YFP cells. Whole-transcriptome microarray hybridization analysis of RNA expression levels in U2OS YFP cells grown for 48 hours as monocultures (YFP_mono_) or as 1:1 co-cultures with WT (YFP_co-Wt_) or R1 (YFP_co-R1_) cells. Expression levels changes were compared as indicated by 2-way ANOVA (culture condition, replicate). Transcripts displaying a >2-fold change in expression are listed.
(DOCX)

S2 Table Metalloprotease-encoding genes differentially expressed in Wt and YFP cells. Gene Ontology (GO) term enrichment analysis of the 82-gene potential competition determinant list. C = 179; O = 7; E = 0.80; R = 8.79; rawP = 1.41e-05; adjP = 0.0007. C, total genes in the GO category, O, occurrence in subject group, rawP, unadjusted P-value, adjP, false discovery adjusted P value, FC, fold change.
(DOCX)
