# Supplementary figures and images for: Spontaneous Cell Competition in Immortalized Mammalian Cell Lines
Source: PLoS One. 2015 Jul 22;10(7):e0132437. doi: 10.1371/journal.pone.0132437 (PMC4511643; doi:10.1371/journal.pone.0132437)

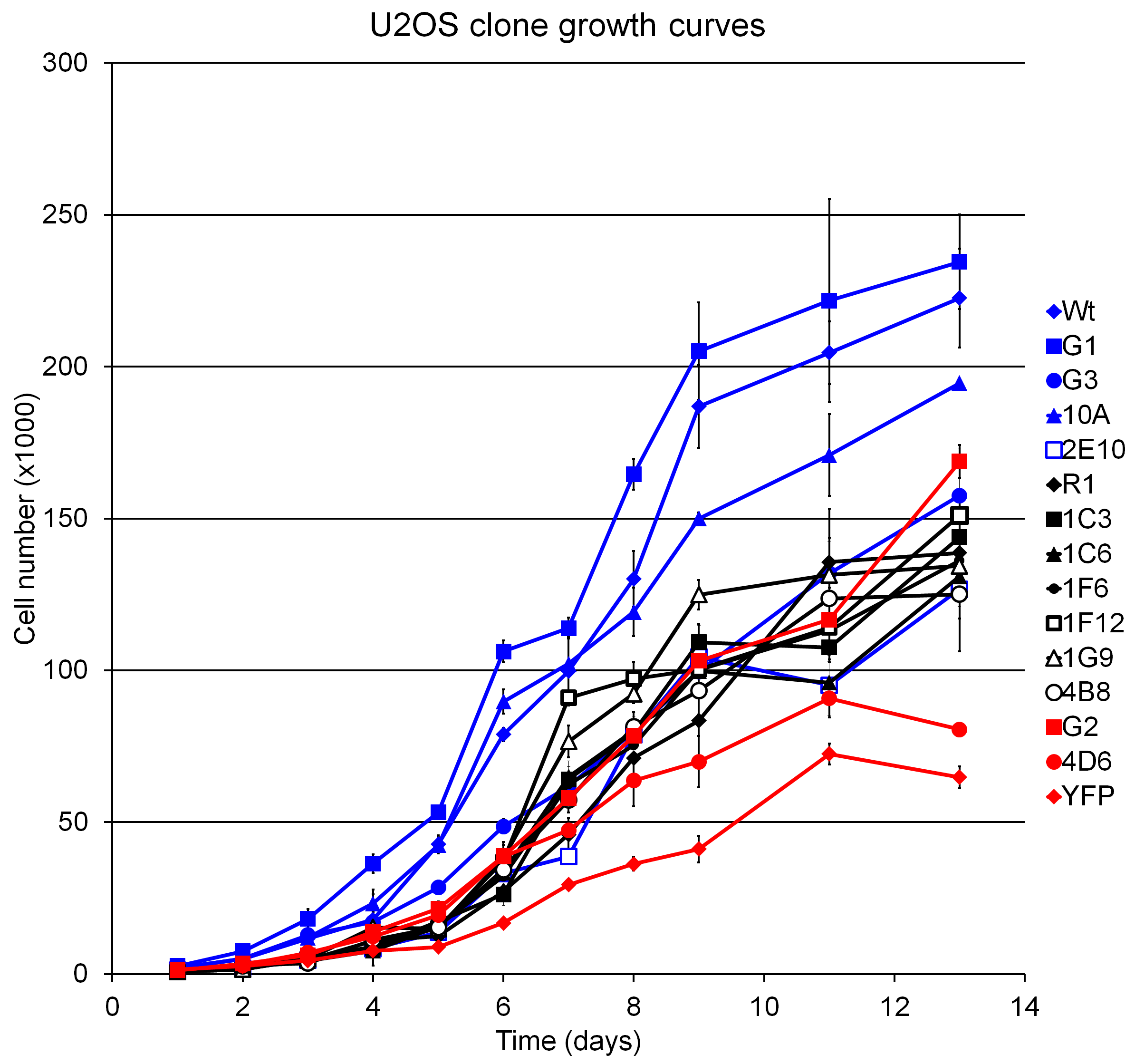

Supplement: S1 Fig — Growth curves of U2OS clones identified in the U2OS screen. Clones are grouped according to the outcome of co-culture with Wt, R1, and YFP cells (see Fig 3). High-fitness clones are shown in blue. They do not compete with Wt and behave as winners in the presence of R1. Clones shown in black behave as losers in the presence of Wt and do not compete with R1. Low-fitness clones, in red, behave as loser in presence of either Wt or R1. (TIF) [file pone.0132437.s001.tif]

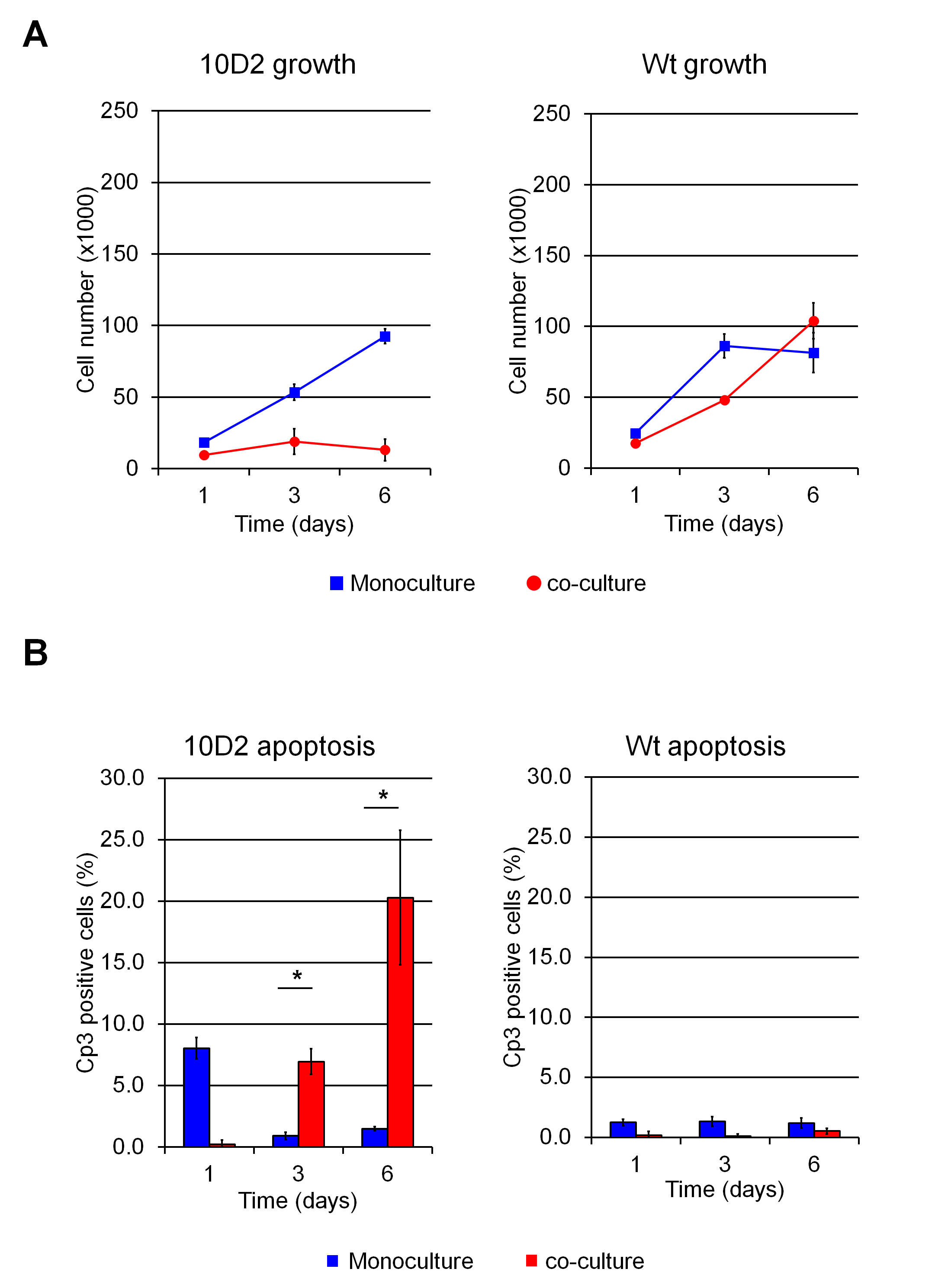

Supplement: S2 Fig — (A) Growth curves of wild-type MDCK cells and H2B-GFP transfectant, single-cell derived 10D2 clone in mono- or co-culture. (B) Apoptosis quantification by Cp3-IF. 10D2 cells undergo increased apoptosis resulting in 10D2 cell number decrease in 10D2:Wt co-cultures. (TIF) [file pone.0132437.s002.tif]

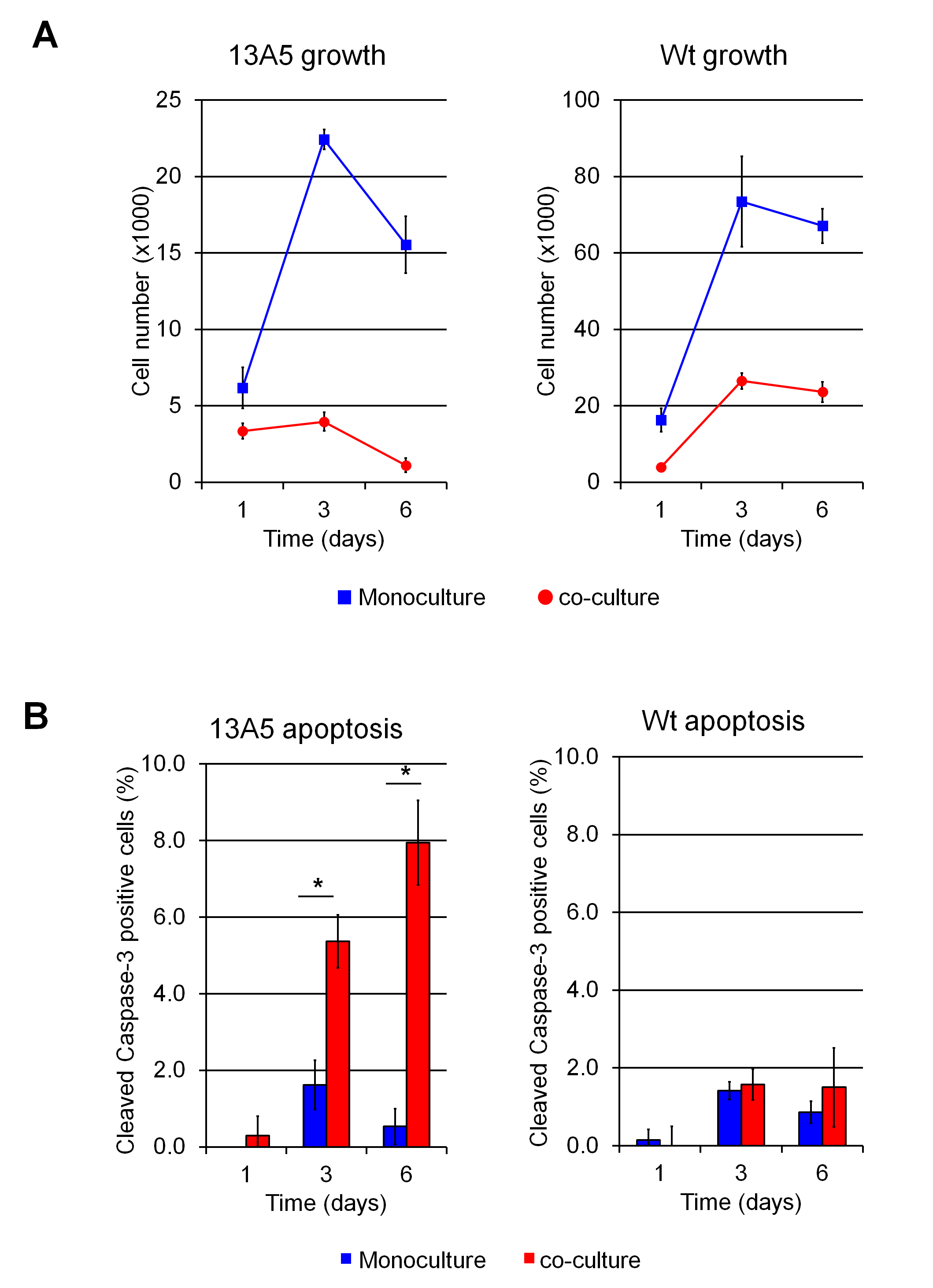

Supplement: S3 Fig — (A) Growth curves of wild-type 3T3 fibroblasts and H2B-GFP stable transfectant, single cell derived 13A5 clone cells in mono- or co-culture. (B) Apoptosis quantification by Cp3-IF. 13A5 cells are viable in mono-culture but display increased apoptosis in the presence of Wt cells and are progressively eliminated from the co-cultures. (TIF) [file pone.0132437.s003.tif]

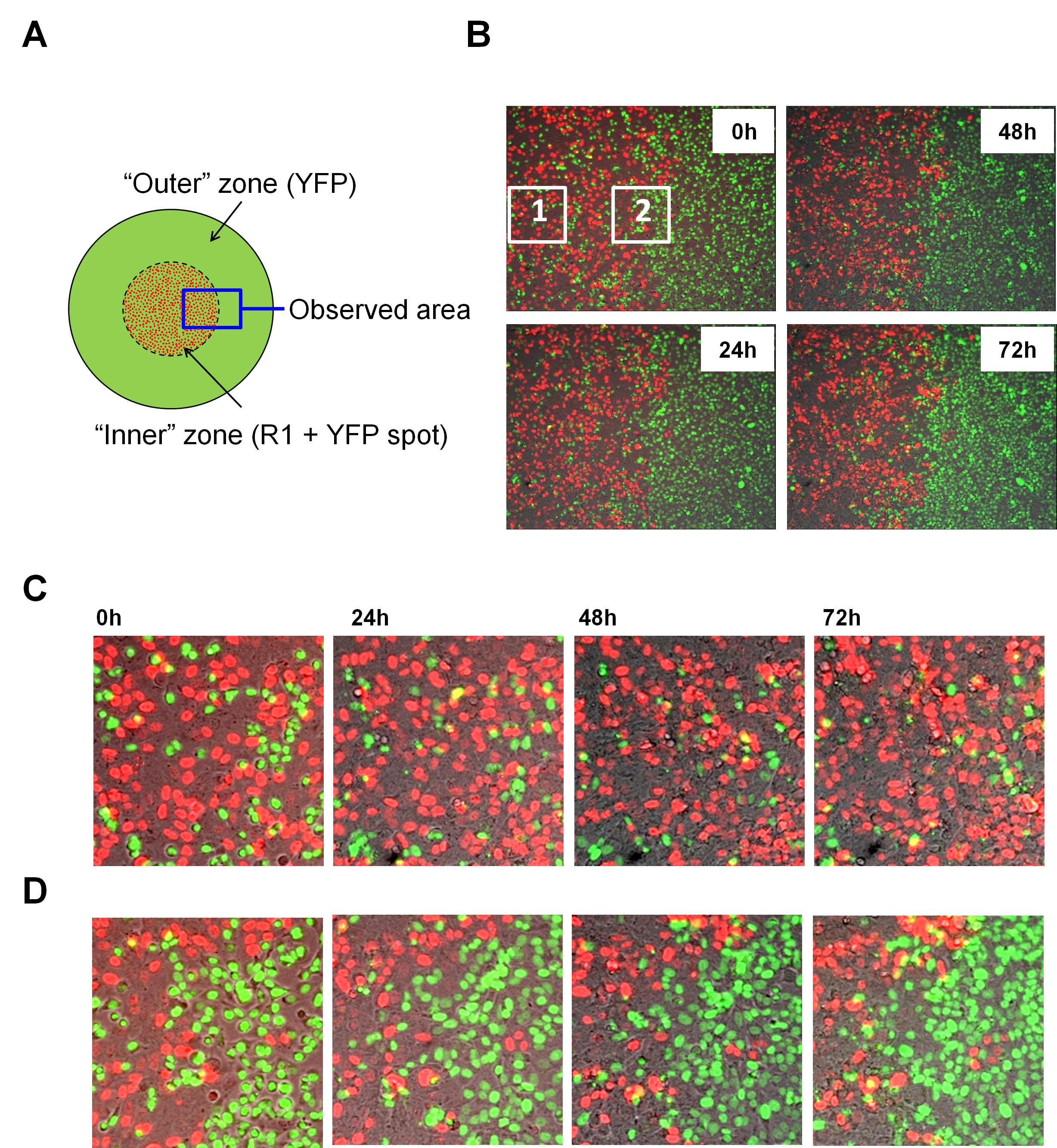

Supplement: S4 Fig — (A) Schematic representation of spotted U2OS-R1 cells overlaid with U2OS-YFP cells. (B) Time-course micrographies of spot cultures. Detail of areas labeled as “1” and “2” is presented in (C) and (D), respectively. Progressive elimination of U2OS-YFP cells inside the R1 spot can be observed, but the spot boundary remains in place, indicating that YFP cells outside are still increasing in number. (TIF) [file pone.0132437.s004.tif]

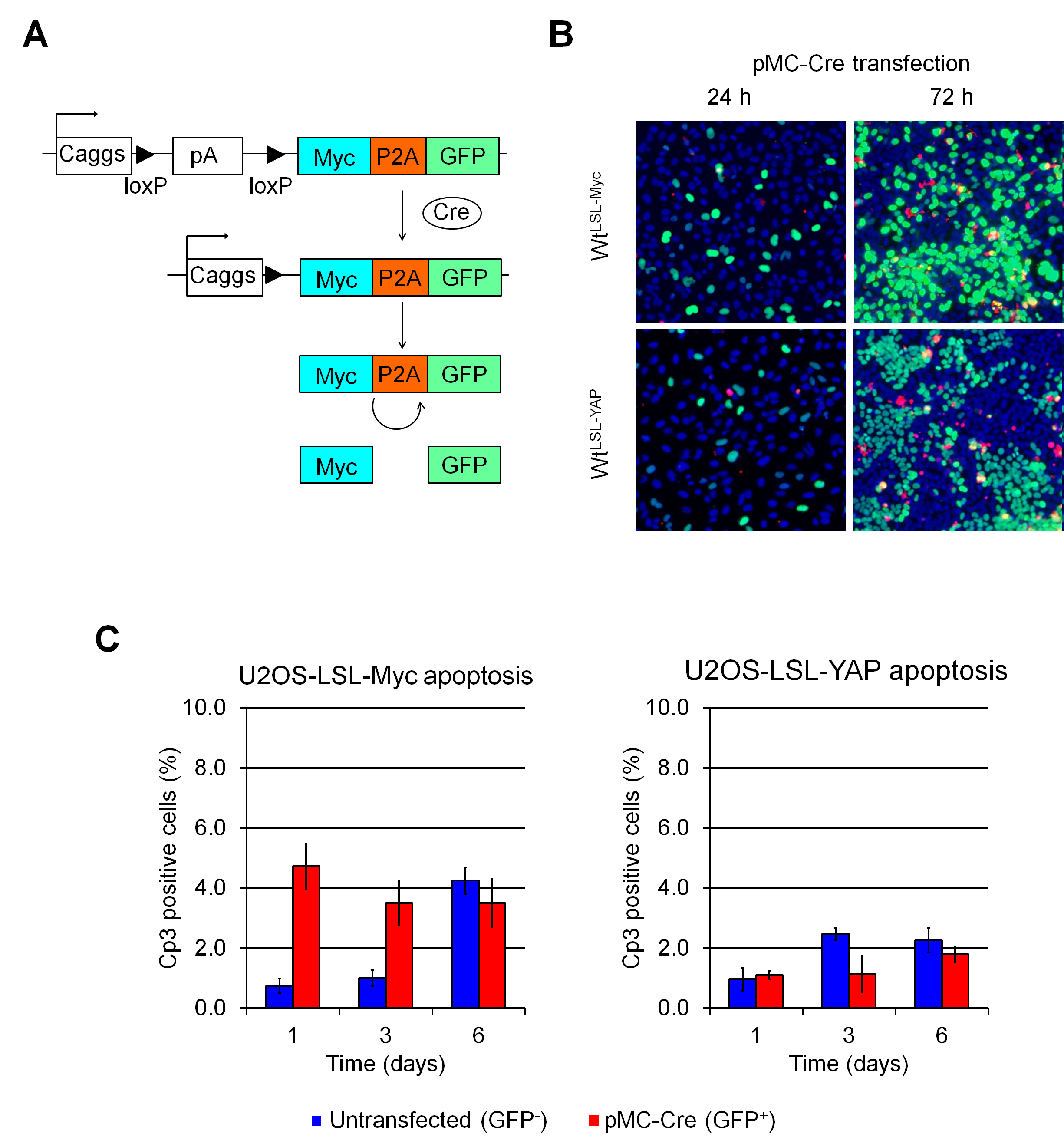

Supplement: S5 Fig — (A) Schematic representation of the inducible LSL-Myc-GFP expression cassette. Cre-mediated recombination results in excision of the SV40 polyadenylation signal (pA), placing the Myc-2A-GFP coding sequence under control of the CMV-actin hybrid promoter (Caggs). The Myc and GFP sequences are separated by the picornavirus 2A self-cleaving sequence, resulting in bi-cistronic MYC and GFP expression. The LSL-YAP-GFP expression cassette was similarly constructed by replacing the Myc coding sequence with that of the constitutive YAPS117A mutant. (B) Aspect of LSL-Myc-GFP and LSL-YAP-GFP stable transfectant U2OS cells transiently transfected with a Cre expression vector (pMC-Cre). Cp3 IF quantification of apoptosis is shown in (C). Apoptosis rates in untransfected cells is similar or lower than that observed in Cre-transfected, Myc/YAP-GFP expressing cells, indicating that Myc and YAP expression does not confer supercompetitor status to U2OS cells. (TIF) [file pone.0132437.s005.tif]

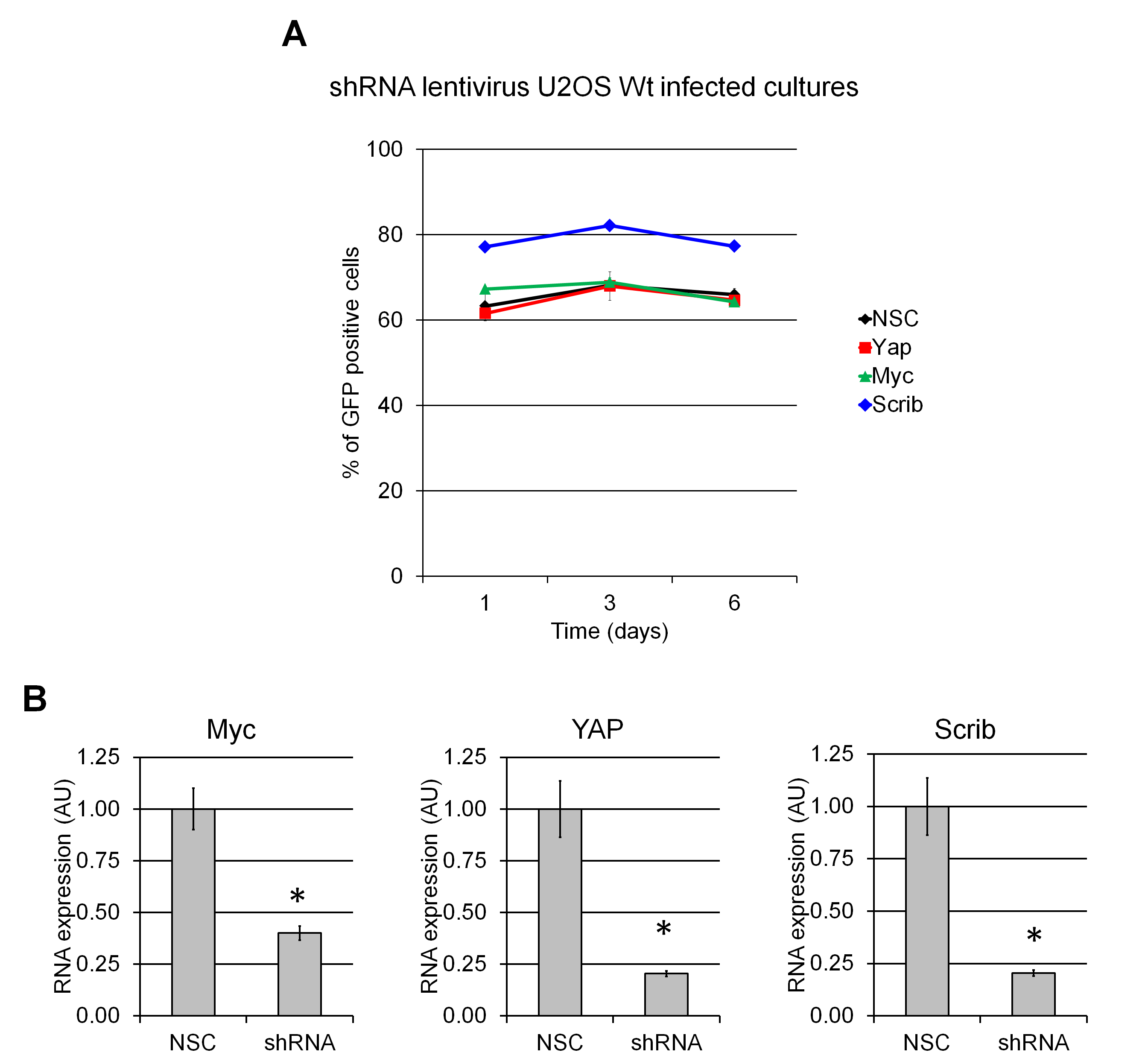

Supplement: S6 Fig — (A) Growth curves of lentivirus-transduced U2OS cells expressing shRNAs directed against Myc, YAP, or Scribbled; cultured alone or alongside Wt cells. shRNA-expresing cells are recognized by means of a GFP expression cassette contained in the lentiviral shRNA vector (not shown). None of these shRNAs induced cell competition. (B) qPCR analysis of gene expression showing reduced Myc, YAP, and Scribbled RNA levels in shRNA-expressing cells. *: p<0.001 (Student’s t-test). (TIF) [file pone.0132437.s006.tif]

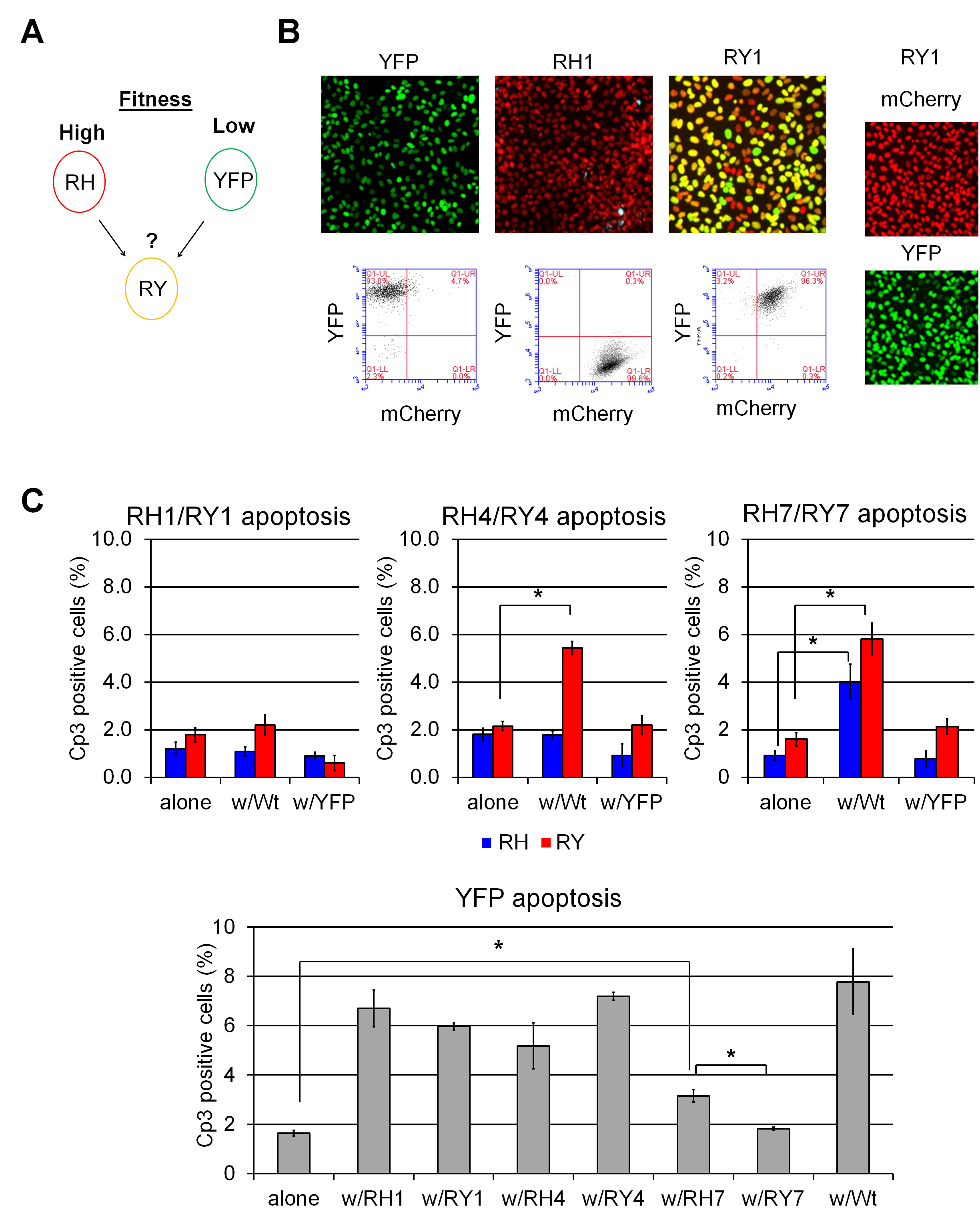

Supplement: S7 Fig — (A) U2OS “winner-loser” cell fusion experimental design. Single-cell derived, H2B-mCherry, hygror stable transfectant clones (RH) were fused to YFP (neor) cells to generate RY cell lines. RY cell fitness levels were then tested by co-culturing RY cells with either Wt or YFP cells. (B) Fluorescence micrographs and flow cytometry profiles of U2OS RY1 cells 4 days after fusion. The result is typical of observed cell fusion outcomes. Separate images of RY1 cell mCherry and YFP fluorescence are shown on the far right. Flow cytometry profiles showing YFP and mCherry co-expression in >95% of RY1 cells are displayed at the bottom. (C) Cp3 IF apoptosis analysis in 72 hour Wt, RH, RY and YFP cultures. Results shown are representative of 3 independent fusion experiments for each RH:YFP pairing. All three RH cell lines behave as “winners” in the presence of YFP cells. RH1:YFP fusion results in a fully “winner” line indicating that YFP “loser” status is rescued and therefore results from loss of function. However, RH4:YFP results in partial rescue and RH7:YFP fusion results in no rescue at all, suggesting that multiple factors determine U2OS cell “fitness”. (TIF) [file pone.0132437.s007.tif]

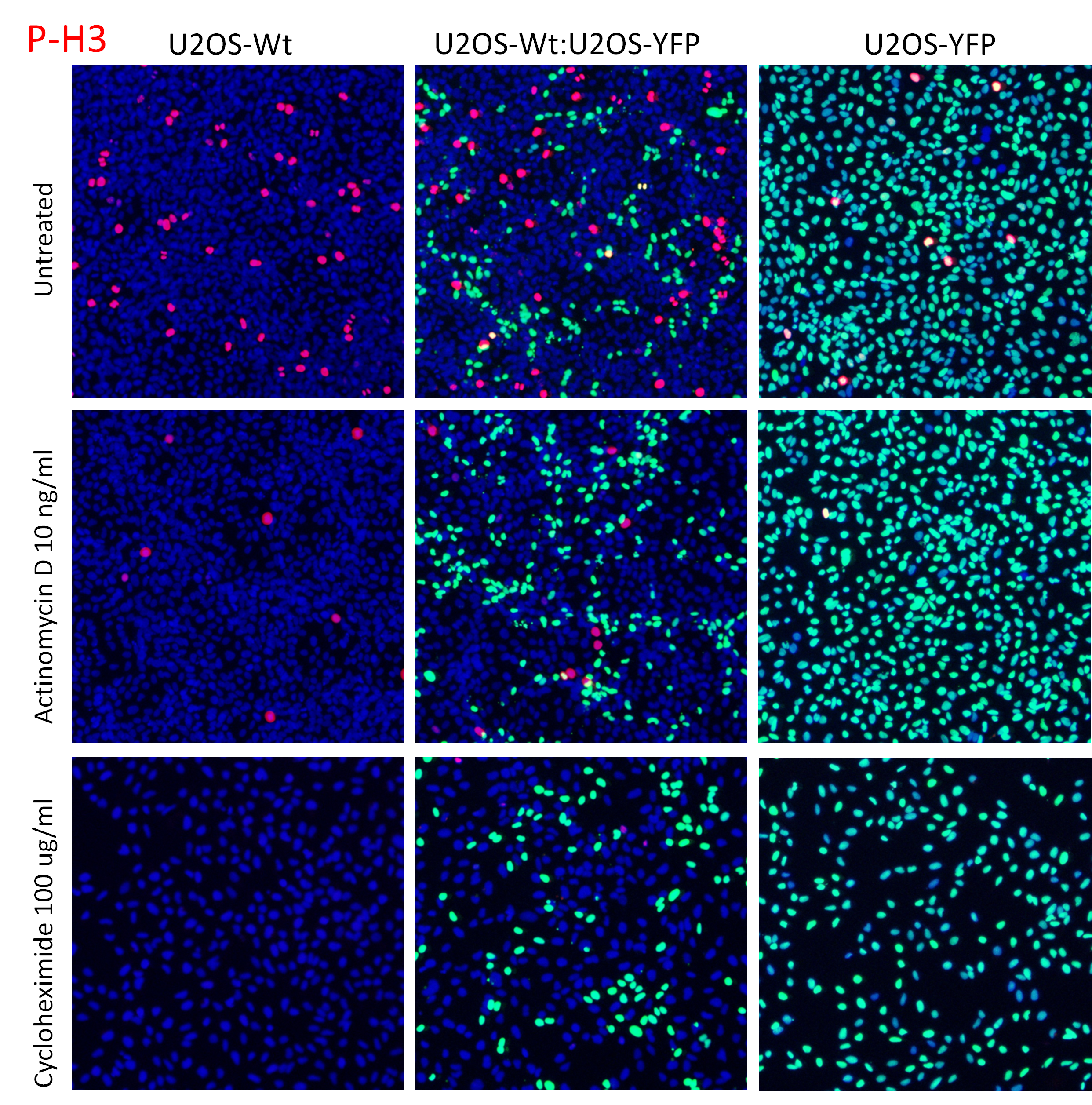

Supplement: S8 Fig — (A) Venn diagram distribution of transcripts displaying a 2-fold or greater expression level difference in Wt vs. R1 vs.YFP cells by whole transcriptome microarray analysis (see Fig 4 for detail of experimental design). (B) Hierarchical clustering of expression profiles of the 422 transcripts displaying a greater than 2-fold expression level change in the Wt vs YFP comparison. Three clusters matching the profile Wt>R1>YFP and 2 clusters matching YFP>R1>Wt were identified (a total of 82 transcripts), suggesting that these transcripts could play a role as “fitness” determinants. (TIF) [file pone.0132437.s008.tif]

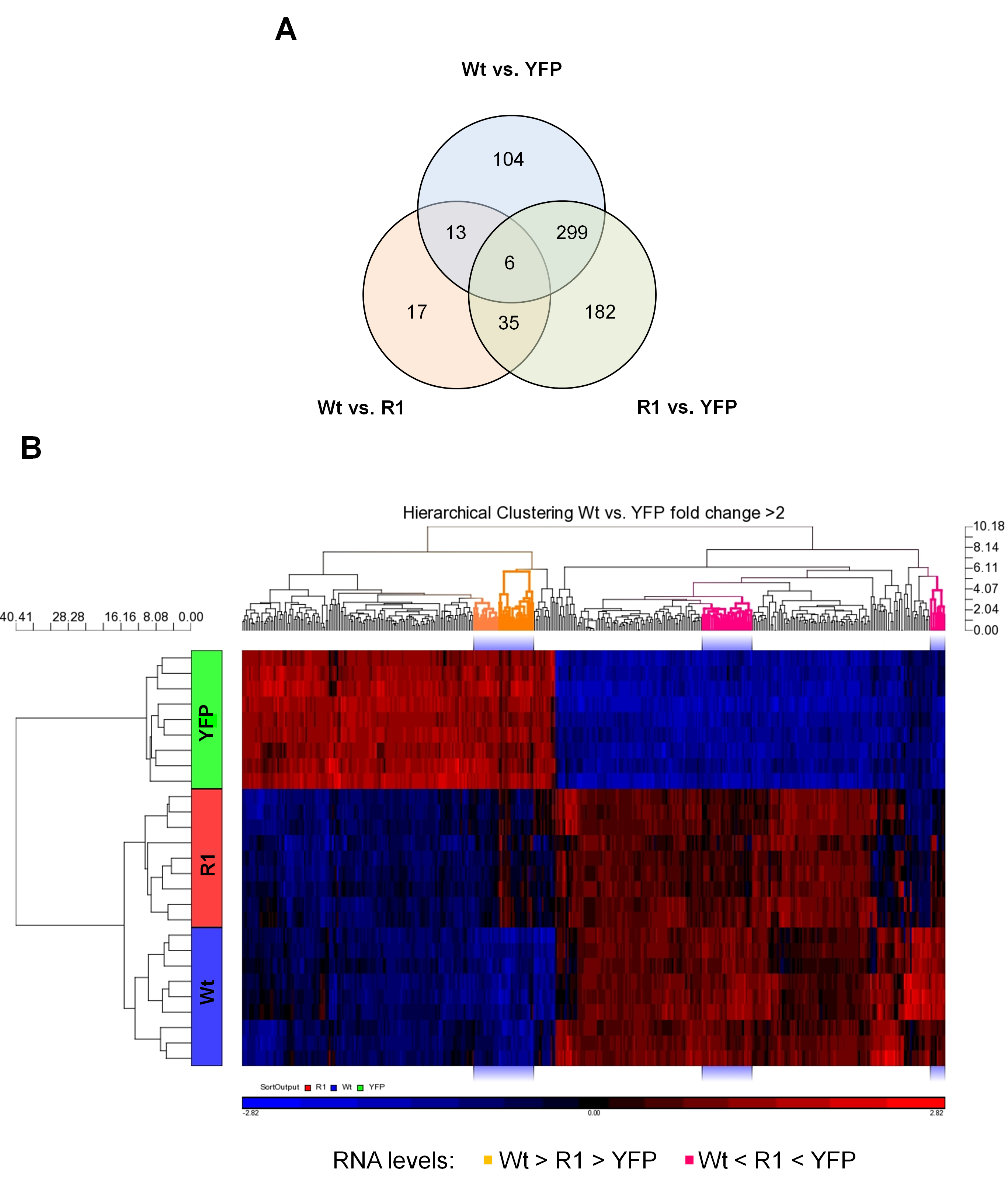

Supplement: S9 Fig — (A) PH3 IF (red) of 48 h U2OS cell cultures grown in presences or absence of actinomycind D or cycloheximide as indicated. Actinomycin D treatment partially inhibits proliferation, suggesting that partial rescue of YFP cell elimination in treated Wt:YFP cultures is due to decreased overall culture growth (See Fig 5). Cycloheximide treatment results in complete proliferation blockade within 48 hours, along with cell competition arrest (Fig 5). (TIF) [file pone.0132437.s009.tif]

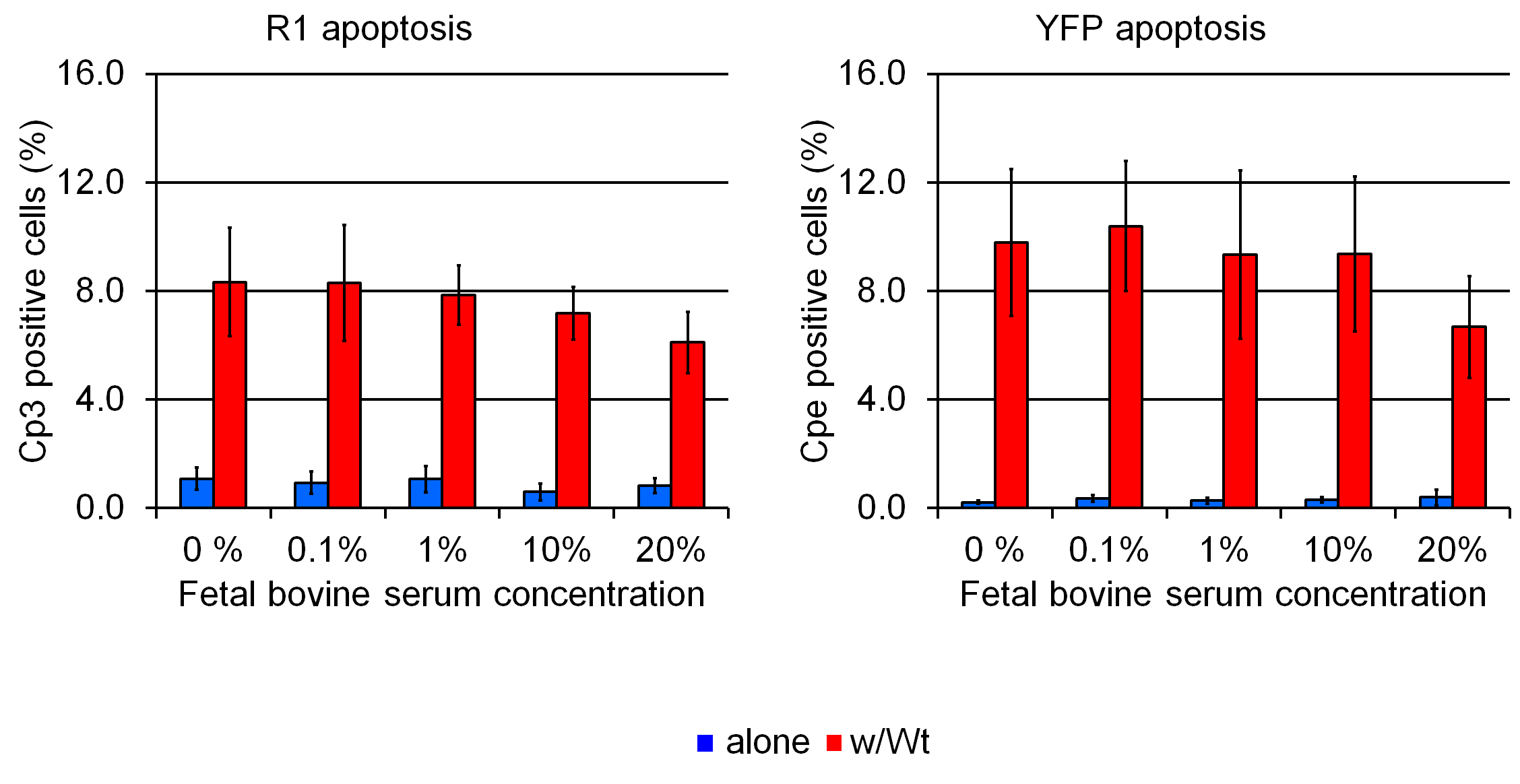

Supplement: S10 Fig — Cp3 IF analysis of apoptosis in 72 hour U2OS cultures grown in medium supplemented with fetal bovine serum at the concentrations indicated. Serum concentration does not affect apoptosis rates in R1 and YFP cells cultured alone or in alongside with Wt cells. (TIF) [file pone.0132437.s010.tif]

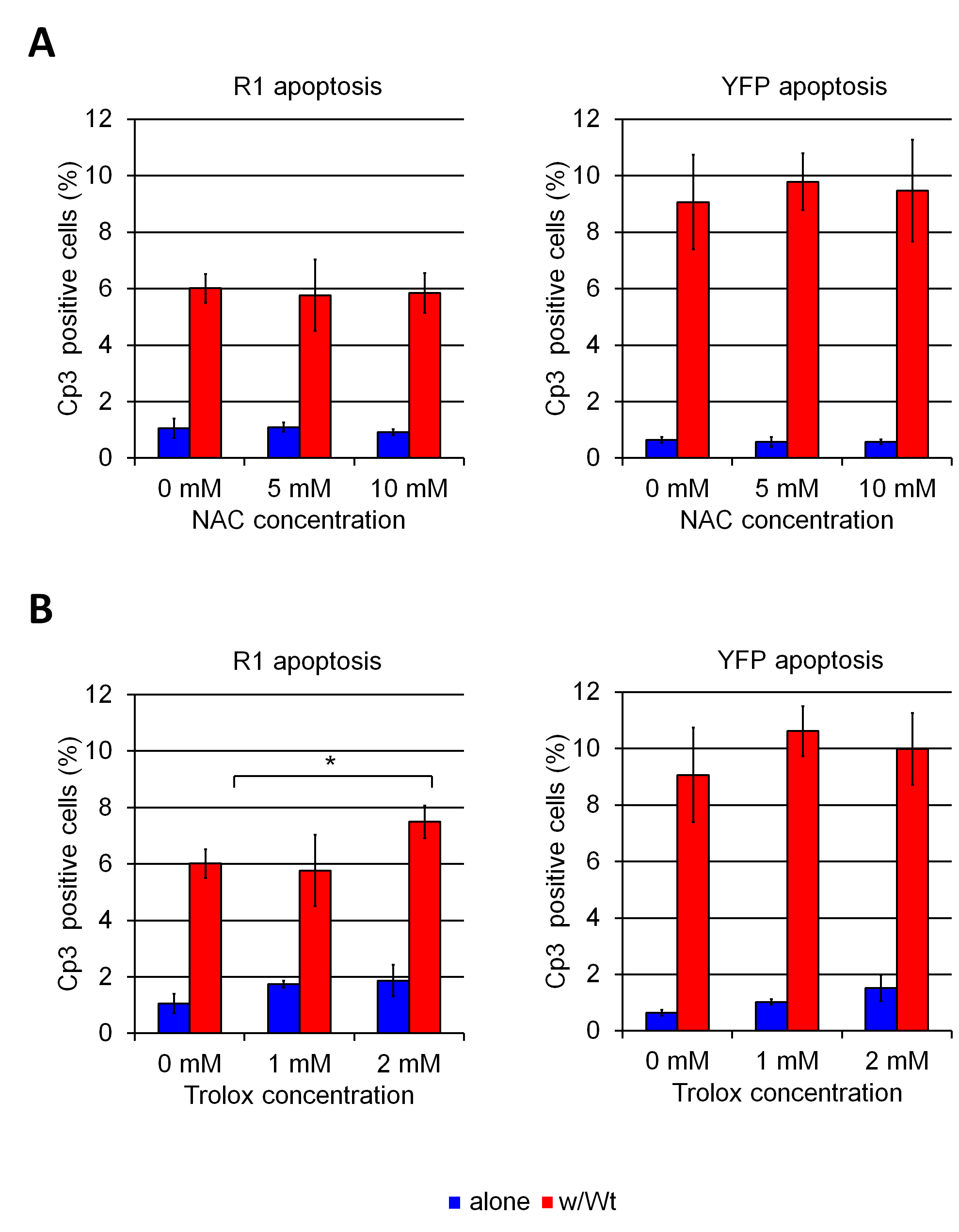

Supplement: S11 Fig — Cp3 IF analysis of apoptosis in 72 h U2OS cultures treated with raical ROS scavengers N-acetyl cysteine (NAC) (A) and 6-hydroxy-2,5,7,8-tetramethylchroman-2-carboxylic acid (Trolox) (B), as indicated. ROS scavengers do not reduce loser cell apoptosis rates in Wt:R1 or Wt:YFP co-cultures. A slight increase is observed in competing R1 cells at the highest Trolox concentration. *:p<0.05 (Student’s t-test). (TIF) [file pone.0132437.s011.tif]
