# Supplementary material for: Spontaneous Cell Competition in Immortalized Mammalian Cell Lines
Source: PLoS One. 2015 Jul 22;10(7):e0132437. doi: 10.1371/journal.pone.0132437 (PMC4511643; doi:10.1371/journal.pone.0132437)
Supplement: S1 Table — Whole-transcriptome microarray hybridization analysis of RNA expression levels in U2OS YFP cells grown for 48 hours as monocultures (YFPmono) or as 1:1 co-cultures with WT (YFPco-Wt) or R1 (YFPco-R1) cells. Expression levels changes were compared as indicated by 2-way ANOVA (culture condition, replicate). Transcripts displaying a >2-fold change in expression are listed. (DOCX) [file pone.0132437.s013.docx]

|  |  |  | YFP_mono_ vs. YFP_co-Wt_ | |  | YFP_mono_ vs. YFP_co-R1_ | |
| --- | --- | --- | --- | --- | --- | --- | --- |
| **RefSeq** | **Symbol** | **Gene** | **Fold-Change** | **p-value** |  | **Fold-Change** | **p-value** |
| NM_001105533 | CPED1 | cadherin-like and PC-esterase domain containing 1 | -2.24 | 1.6E-07 |  | -1.85 | 5.14E-06 |
| BC069094 | OR51B4 | olfactory receptor, family 51, subfamily B, member 4 | -3.44 | 4.9E-08 |  | -2.47 | 2.96E-06 |
| AK291957 | IL13RA2 | interleukin 13 receptor, alpha 2 | -3.49 | 8.4E-08 |  | -2.41 | 7.52E-06 |
| BC105587 | GTSF1 | gametocyte specific factor 1 | -5.64 | 2.5E-09 |  | -3.78 | 9.85E-08 |
| BC136707 | OR51B5 | olfactory receptor, family 51, subfamily B, member 5 | -2.50 | 1.2E-06 |  | -1.76 | 2.75E-04 |
| BC005261 | SLN | sarcolipin | -2.36 | 5.4E-06 |  | -1.79 | 3.67E-04 |
