# Supplementary material for: Spontaneous Cell Competition in Immortalized Mammalian Cell Lines
Source: PLoS One. 2015 Jul 22;10(7):e0132437. doi: 10.1371/journal.pone.0132437 (PMC4511643; doi:10.1371/journal.pone.0132437)
Supplement: S2 Table — Gene Ontology (GO) term enrichment analysis of the 82-gene potential competition determinant list. C = 179; O = 7; E = 0.80; R = 8.79; rawP = 1.41e-05; adjP = 0.0007. C, total genes in the GO category, O, occurrence in subject group, rawP, unadjusted P-value, adjP, false discovery adjusted P value, FC, fold change. (DOCX) [file pone.0132437.s014.docx]

| **GO:0008237 molecular function: metallopeptidase activity** | | |
| --- | --- | --- |
| **Symbol** | **Description** | **FC (Wt/YFP)** |
| TLL2 | tolloid-like 2 | 2.58 |
| ADAMTS1 | ADAM metallopeptidase with thrombospondin type 1 motif, 1 | 2.37 |
| ADAMTSL1 | ADAMTS-like 1 | 2.25 |
| ADAM12 | ADAM metallopeptidase domain 12 | 2.24 |
| MMP3 | matrix metallopeptidase 3 (stromelysin 1, progelatinase) | -2.4 |
| CPE | carboxypeptidase E | -2 |
| FAP | fibroblast activation protein, alpha | -4.45 |
|  | | |
